# Trem2/Syk/PI3K axis contributes to the host protection against *Toxoplasma gondii*-induced adverse pregnancy outcomes via modulating decidual macrophages

**DOI:** 10.1371/journal.ppat.1012543

**Published:** 2024-09-09

**Authors:** Qing Wang, Yining Cao, Songyi Ye, Maoyuan Ding, Wenliang Ge, Yuejin Liang, Jinling Chen

**Affiliations:** 1 Department of Pathogen Biology, School of Medicine, Nantong University, Nantong, Jiangsu, People’s Republic of China; 2 Department of Pediatric Surgery, Affiliated Hospital of Nantong University, Nantong, Jiangsu, People’s Republic of China; 3 Department of Microbiology & Immunology, The University of Texas Medical Branch Galveston, Texas, United States of America; University of Wisconsin Medical School, UNITED STATES OF AMERICA

## Abstract

Decidual macrophages residing at the maternal-fetal interface have been recognized as pivotal factors for maintaining normal pregnancy; however, they are also key target cells of *Toxoplasma gondii* (*T*. *gondii*) in the pathology of *T*. *gondii*-induced adverse pregnancy. Trem2, as a functional receptor on macrophage surface, recognizes and binds various kinds of pathogens. The role and underlying mechanism of Trem2 in *T*. *gondii* infection remain elusive. In the present study, we found that *T*. *gondii* infection downregulated Trem2 expression and that Trem2^-/-^ mice exhibited more severe adverse pregnancy outcomes than wildtype mice. We also demonstrated that *T*. *gondii* infection resulted in increased decidual macrophages, which were significantly reduced in the Trem2^-/-^ pregnant mouse model as compared to wildtype control animals. We further described the inhibited proliferation, migration, and invasion functions of trophoblast cell by *T*. *gondii* antigens through macrophages as an "intermediate bridge", while this inhibition can be rescued by Trem2 agonist HSP60. Concurrently, Trem2 deficiency in bone marrow-derived macrophages (BMDMs) heightened the inhibitory effect of *Tg*Ag on the migration and invasion of trophoblast cells, accompanied by higher pro-inflammatory factors (IL-1β, IL-6 and TNF-α) but a lower chemokine (CXCL1) in *T*. *gondii* antigens-treated BMDMs. Furthermore, compelling evidence from animal models and *in vitro* cell experiments suggests that *T*. *gondii* inhibits the Trem2-Syk-PI3K signaling pathway, leading to impaired function of decidual macrophages. Therefore, our findings highlight Trem2 signaling as an essential pathway by which decidual macrophages respond to *T*. *gondii* infection, suggesting Trem2 as a crucial sensor of decidual macrophages and potential therapeutic target in the pathology of *T*. *gondii*-induced adverse pregnancy.

## Author summary

*T*. *gondii* is a critically important intracellular protozoan that is a major cause of adverse pregnancy outcomes in humans and livestock. The pathology of adverse pregnancy induced by *T*. *gondii* involves the dysfunction of decidual macrophages. Trem2, a functional immune receptor on the surface of decidual macrophages, governs its survival and phagocytosis. Here, we sought to elucidate whether Trem2 signaling is involved in the pathology of *T*. *gondii*-induced adverse pregnancy. We found that *T*. *gondii* infection led to the downregulation of Trem2 expression in macrophages, both *in vivo* and *in vitro*. Trem2^-/-^ mice exhibit more severe adverse pregnancy outcomes after *T*. *gondii* infection. We further described the inhibited proliferation, migration, and invasion functions of trophoblast cell by *T*. *gondii* antigens through macrophages as an "intermediate bridge", while this inhibition can be rescued by Trem2 agonist HSP60. In parallel, Trem2 deficiency in BMDMs heightened the inhibitory effect of *Tg*Ag on the migration and invasion of trophoblast cells. Our work uncovers an important factor involved in the pathological mechanism of *T*. *gondii*-induced adverse pregnancy, and research on Trem2 may provide new preventive and therapeutic targets for toxoplasmosis.

## Introduction

*Toxoplasma gondii* (*T*. *gondii*) infects over 30% of the global population, contributing to more than 2 million cases of congenital toxoplasmosis annually. Therefore, toxoplasmosis is a significant global public health concern [1,2]. *T*. *gondii* is a prevalent obligate intracellular protozoan that can disseminate to the placenta, infect maternal leukocytes, invade uterine decidua and trophoblast cells, and induce intense immunopathology in the placenta, ultimately leading to fetal demise, miscarriage, and various other adverse outcomes [3,4]. The pathology of adverse pregnancy outcomes involves the disruption of the immune micro-environment at the maternal-fetal interface, which comprises a significant number of decidual immune cells, primarily macrophages, natural killer (NK) cells, T cells and dendritic cells [5–7]. Decidual macrophages, accounting for approximately 20%-25% of decidual immune counterparts, are crucial regulators in placenta formation, embryo implantation, development and delivery. By secreting cytokines and chemokines, decidual macrophages promote vascular remodeling and trophoblast cell migration and invasion. The crosstalk between decidual macrophages and trophoblast cells significantly contributes to the maintenance and establishment of a normal pregnancy [8–10]. Additionally, decidual macrophages serve as targets for numerous pathogens at the maternal-fetal interface, activating various immune signals as a defense mechanism against these invaders (e.g., *T*. *gondii*, Zika virus (ZIKV), etc.). However, they can also release a diverse array of pro-inflammatory and anti-inflammatory factors, leading to immune imbalances at the maternal-fetal interface [11,12]. *T*. *gondii* infection has been demonstrated to alter the quantity and function of decidual macrophages [13]. Adoptive transfer of decidual macrophages partially improved *T*. *gondii*-induced adverse pregnancy outcomes, as evidenced by the increased fetal weight and the decreased rate of abnormal fetuses, further emphasizing the importance of decidual macrophages in maintaining normal pregnancy [6]. Severe pregnancy outcomes accompanied with blunted decidual macrophage responses to *T*. *gondii* are evident in mice deficient for B7 homolog 4 (B7-H4), T cell immunoglobulin domain and mucin domain-3 (Tim-3), and human leukocyte immunoglobulin-like receptor subfamily B member 4 (LILRB4) [6,13,14]. However, those immunoinhibitory molecules are not predominantly expressed on decidual macrophages. Hence, the functional molecules and associated signaling pathways in decidual macrophages during *T*. *gondii* infection remain elusive.

Triggering receptor expressed on myeloid cells 2 (Trem2), a one-way transmembrane immune receptor, is specifically expressed on macrophages in adipose tissue, adrenal gland, and placenta, according to single-cell RNA sequencing (scRNA-seq) analysis [15]. Upon activation by its ligands, Trem2 signals mainly through the associated articulatory protein DNAX activating protein 12 (DAP12), which recruits and activates the spleen tyrosine kinase (Syk) via the phosphorylation of its immunoreceptor tyrosine-based activation motif (ITAM), leading to the activation of downstream effector molecules including phospholipase C-gamma-2 (PLCγ2), mammalian target of rapamycin (mTOR), phosphoinositide 3-kinase (PI3K), and mitogen-activated protein kinase (MAPK) [16,17]. Trem2 is also implicated in various pathogenic infections due to its ability to recognize and bind pathogens, subsequently, facilitating innate immune responses against pathogens (e.g., Syk-dependent phagocytosis) [18–20]. Notably, the study by the injection of *T*. *gondii* cysts in 5×FAD mice with Alzheimer’s pathology revealed that chronic *T*. *gondii* infection recruited mononuclear macrophages with intermediate and high expression of Trem2 to the central nervous system, suggesting the unique anti-inflammatory and phagocytic effects of Trem2 in *T*. *gondii* infection [21]. In addition, Syk and PI3K, the downstream signaling of Trem2, are also activated during host immunopathology in the responses to *T*. *gondii* infection [22,23]. Similarly, Trem2 also possesses potential immunosuppressive activity [24], and has been demonstrated to be specifically expressed on placental macrophages [15,25]. Notably, a decrease in Trem2^+^ macrophages has been observed in the preeclampsia (PE) placentas [26]. Based on these findings, we hypothesize that Trem2 signaling plays a central role in the pathological mechanism of *T*. *gondii*-induced adverse pregnancy outcomes through its effects on decidual macrophages.

To test our hypothesis, we established *T*. *gondii*-infected wildtype and Trem2^-/-^ pregnant mouse models. We found that *T*. *gondii* infection inhibited surface Trem2 expression on decidual macrophages and altered macrophage functions. Importantly, deficiency of Trem2 resulted in the aggravated adverse pregnancy outcomes during *T*. *gondii* infection. We further elucidated the pathological mechanisms of Trem2 and downstream Syk/PI3K signaling involved in the induction of adverse pregnancy outcomes by *T*. *gondii* infection. Our *in vitro* study demonstrated that *T*. *gondii* can regulate the migration, invasion, and proliferation of trophoblast cells by affecting decidual macrophages. Taken together, Trem2 is a key sensor of decidual macrophages in pathological pregnancy and might be a potential therapeutic target to adverse pregnancies in *T*. *gondii* infection.

## Results

### *T*. *gondii* infection down-regulates Trem2 expression on decidual macrophages in pregnant mice

**Fig 1:**
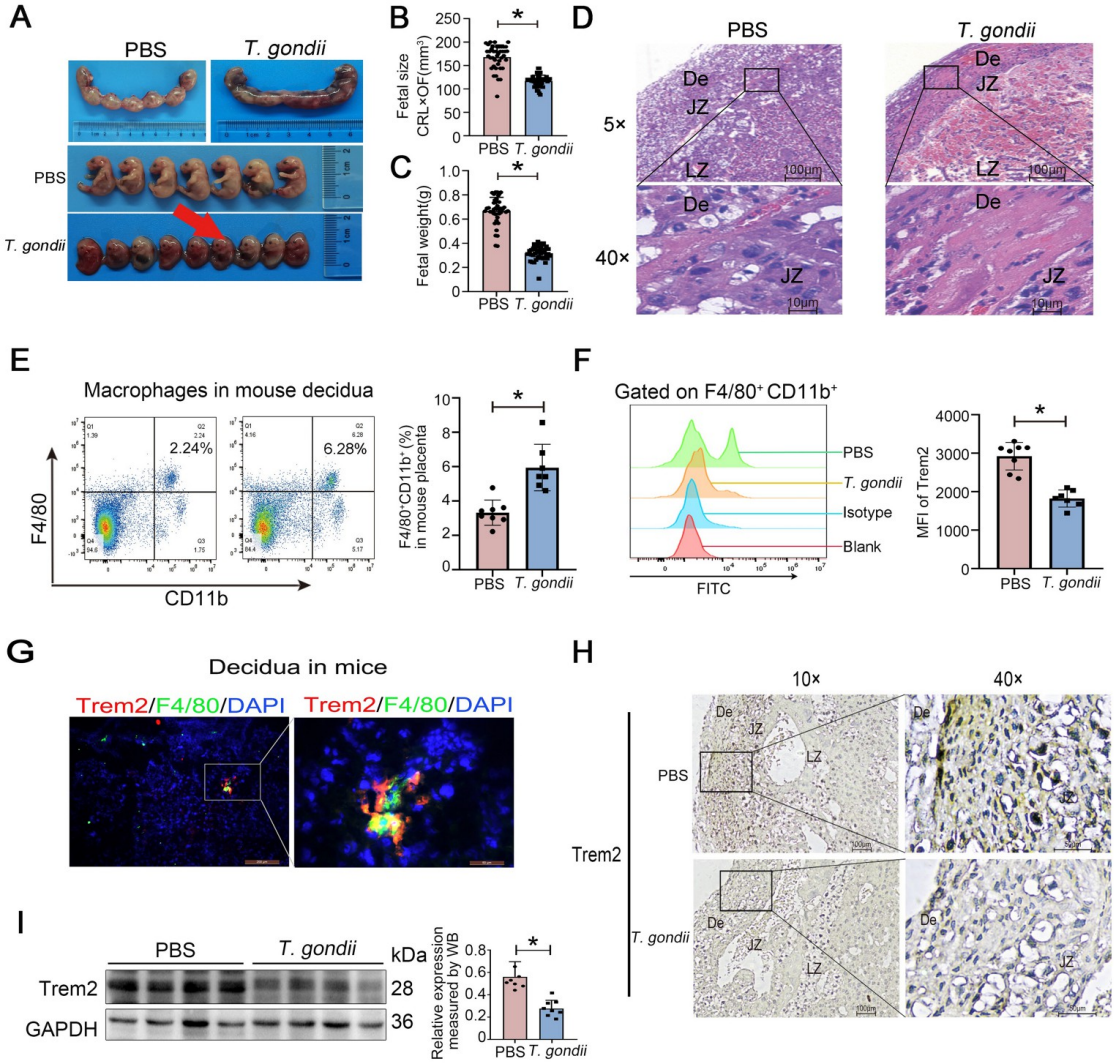
*T*. *gondii* infection down-regulates Trem2 expression on decidual macrophages in pregnant mice. (A) Representative image of the placentas and fetuses of wildtype mice at G 17.5 infected with *T*. *gondii*. Red arrow indicated fetal demise. (B and C) Fetal development assessed by fetal size (CRL×OF) and fetal weight. Each data points represent individual fetuses (n = 5–7 mice). CRL: crown-rump length; OF: occipito-frontal diameter. (D) Representative HE staining showing pathological features of mouse placentas. The normal mouse placental structure is composed of decidua zone (De), junctional zone (JZ), and labyrinth zone (LZ). Pathological necrosis in wildtype mice infected with *T*. *gondii* mainly occurs in the De. (E) Representative flow cytogram of CD11b^+^ F4/80^+^ decidual macrophages, comparing decidual macrophage proportions in normal or infected placentas in mice. The placentas of each mouse were divided into three groups for technically repeated experiments. Data point represents the placenta of a single pregnant mouse (n = 7–8 mice). (F) Representative MFI of Trem2 on the surface of CD11b^+^ F4/80^+^ decidual macrophages in normal or infected placentas in mice. The placentas of each mouse were divided into three groups for technically repeated experiments. Data point represents the placenta of a single pregnant mouse (n = 7–8 mice). MFI: mean fluorescence intensity. (G) Decidual macrophages were labeled with F4/80, and the colocalization between Trem2 (red) and F4/80 (green) was imaged by immunohistofluorescence. (H) Immunohistochemistry of normal and infected mouse placentas, which was immunostained with anti-Trem2 and counterstained with hematoxylin. (I) Immunoblot of Trem2 expression and statistical analysis on normal and infected mouse placentas. Each data point represents the placenta of an individual pregnant mouse (n = 8 mice). Data were presented as mean ± SD. Statistical analysis was conducted using two-tailed unpaired Student’s *t*-test (B, C, E, F and I). *: *P* < 0.05.

A mouse model of pregnancy with *T*. *gondii* infection was constructed by intraperitoneally (i.p.) injection of *T*. *gondii* tachyzoites at 8.5 days of gestation (G8.5). Significant fetal death and developmental delay were observed in the dams of *T*. *gondii*-injected mice at G17.5, as shown in Fig 1A. By measuring fetal weight and size, we found that *T*. *gondii* infection also resulted in significant fetal growth restriction (Fig 1B and 1C). The placenta serves as a functional interface for material exchange between the mother and fetus, and its decidual structure provides nutritional support to the embryo and protects the embryo from attack by maternal immune cells during pregnancy, playing a critical role on the normal development of the fetus [27–29]. However, *T*. *gondii* can cross and disrupt the placenta during pregnancy. We therefore determined whether pathological changes occurred in the mouse placenta after *T*. *gondii* infection by HE staining. Our results exhibited obvious necrosis in the decidual area (De) of the *T*. *gondii*-infected mouse placentas (Fig 1D), indicating the destruction of the placental structure and impaired substance exchange caused by *T*. *gondii*.

As an extremely abundant immune cell population at the maternal-fetal interface, decidual macrophages not only participate in the innate immune responses against pathogens, but also actively maintain normal pregnancy [6,30]. Trem2 signaling regulates a variety of physiological functions in macrophages, including cell survival, metabolic regulation, and anti-inflammatory responses. More importantly, Trem2 signaling pathway is also critical for macrophages in responses to a wide range of pathogens. Concurrently, given Trem2’s appreciated centrality in macrophage function [31], we asked whether *T*. *gondii* infection affects decidual macrophages through Trem2 signaling. To test this, we analyzed CD11b^+^ F4/80^+^ decidual macrophages by flow cytometry and found that the percentage of cells was significantly increased in the *T*. *gondii*-infected group compared with the uninfected group (Fig 1E). To validate the impact of *T*. *gondii* infection on Trem2 expression, we next assessed Trem2 expression on decidual macrophages. Our results showed that Trem2 expression was down-regulated on decidual macrophages by *T*. *gondii* infection (Fig 1F). Thus, *T*. *gondii* not only impairs the normal structure of the placenta may also alter immune micro-environment by regulating Trem2 expression on decidual macrophages.

Decidual macrophage is a critical regulator in defense against pathogens, notably, *T*. *gondii*, Zika virus, and human immunodeficiency virus [13,32]. The dysfunction of decidual macrophages is highly related to implantation failure, fetal rejection and vascular development defects. Trem2 acts as a functional immunomodulatory receptor on macrophages; however, its role in *T*. *gondii* infection is unclear. To identify whether Trem2 is a key factor related to decidual macrophage function during *T*. *gondii*-induced adverse pregnancy, we first evaluated Trem2 protein in wildtype mouse placenta tissues through immunohistofluorescence and confirmed the Trem2 expression on decidual macrophages (Fig 1G). Similar results were observed by immunohistochemical staining of placental tissues, where Trem2 was expressed in the De of mouse placentas. Comparing the staining results of the placentas between normal and infected mice, we found that Trem2 expression was decreased in the placentas of mice infected with *T*. *gondii* on G8.5, as evidenced by a greatly reduced area of yellow-brown positive areas (Fig 1H). To further validate these findings, we extracted placental tissue proteins for immunoblot assay. The result analysis confirmed that *T*. *gondii* infection caused the down-regulation of Trem2 expression (Fig 1I). These experimental results indicate that *T*. *gondii* infection may impair the function of decidual macrophages by inhibiting Trem2 signaling.

### Trem2 deficiency aggravates *T*. *gondii*-induced adverse pregnancy

**Fig 2:**
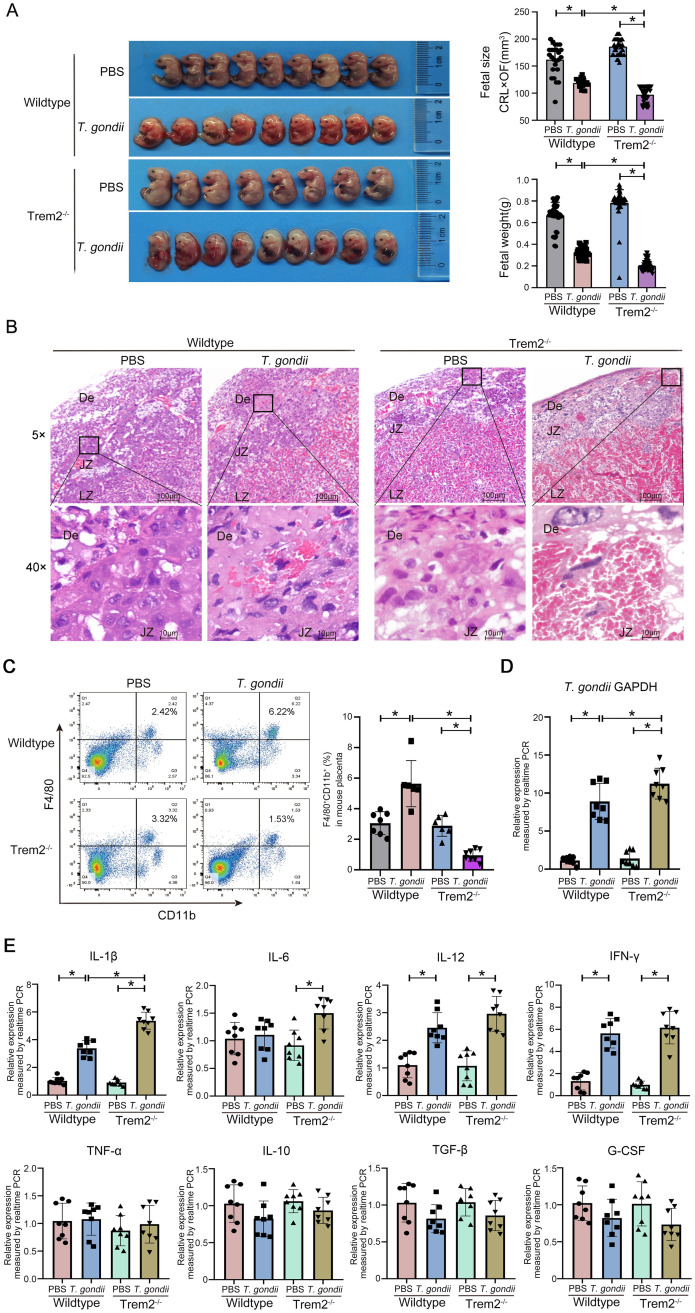
*T*. *gondii* infection aggravates adverse pregnancy outcomes in Trem2^-/-^ mice. (A) Representative images of fetuses at G17.5 from wildtype and Trem2^-/-^ pregnant mice with or without *T*. *gondii* infection. Fetal development was assessed by fetal size (CRL×OF) and fetal weight. Data point represents the placenta of a single pregnant mouse (n = 5–7 mice). (B) Representative HE staining shows the pathological characteristics of wildtype and Trem2^-/-^ mouse placentas, with hemorrhage and necrosis lesions present in the De of the infected group. (C) Representative flow cytogram of CD11b^+^ F4/80^+^ decidual macrophages, comparing decidual macrophage proportions in wildtype and Trem2^-/-^ mouse placentas with or without *T*. *gondii* infection. The placentas of each mouse were divided into three groups for technically repeated experiments. Data point represents the placenta of a single pregnant mouse (n = 6–8 mice). (D) *T*. *gondii* burden in the placentas of wildtype and Trem2^-/-^ mice was measured by real-time PCR (n = 8 mice). (E) mRNA levels of IL-1β, IL-6, IL-10, IL-12, IFN-γ, TNF-α, TGF-β and G-CSF in the mouse placentas were assayed by real-time PCR (n = 8 mice). Data were presented as mean ± SD. Statistical analysis was conducted using one-way ANOVA with Tukey’s multiple comparisons test (A, C, D and E). *: *P* < 0.05.

To investigate the role of Trem2 in adverse pregnancy induced by *T*. *gondii* infection, we constructed Trem2^-/-^ mice, which had been previously genotyped [33]. We then used the same method to establish a pregnancy model of Trem2^-/-^ mice and compared the pregnancy outcomes between Trem2^-/-^ and wildtype mice before and after *T*. *gondii* infection. All fetuses underwent a consistent macroscopic assessment by fetal size and weight. Our results demonstrated the comparable fetal size and weight between pregnant wildtype and Trem2^-/-^ mice without *T*. *gondii* infection. However, *T*. *gondii* infection resulted in more severe adverse pregnancy outcomes in Trem2^-/-^ mice compared with wildtype mice, as evidenced by the reduced fetal size and weight (Fig 2A). This finding suggested the critical role of Trem2 on alleviating *T*. *gondii*-induced adverse outcomes. Additionally, HE staining manifested that the placentas of the infected Trem2^-/-^ mice also suffered pathological damage, accompanied by obvious bleeding and necrosis (Fig 2B). Next, we sought to explore whether Trem2 deficiency alters the quantity and function of placental immune cells in *T*. *gondii*-induced adverse pregnancy. We analyzed the decidual macrophages, NK cells and T cells in the placenta by flow cytometry and found that the percentages of all cell populations were comparable between wildtype and Trem2^-/-^ mice without *T*. *gondii* infection (Fig 2C and S1 Fig). In the context of *T*. *gondii* infection, wildtype and Trem2^-/-^ mice exhibited a significant increase in decidual macrophages, NK cells and T cells. However, the percentage and absolute number of decidual macrophages in infected Trem2^-/-^ mice were noticeably declined, whereas the numbers of NK cells and T cells in infected Trem2^-/-^ mice were not significantly changed as compared with infected wildtype mice (Fig 2C and S1 Fig). Our results may suggest that Trem2 signals contribute to immune responses of decidual macrophages, but not NK and T cells in *T*. *gondii* infection. It is reported that Trem2 can promote the survival and recruitment of macrophages at pathological sites under pathological conditions such as tissue damage and inflammation [15,34]. Our results may suggest that Trem2 may play a marginal role in maintaining decidual macrophage number and embryonic development during the homeostasis of normal pregnancy; conversely, Trem2 signaling may trigger the responses of decidual macrophages to *T*. *gondii* invasion and protect the host from adverse pregnancy outcomes.

Trem2 has been shown to be involved in host resistance to certain parasites (e.g., *Plasmodium berghei*) [33,35]. To investigate the role of Trem2 in *T*. *gondii* infection, parasite burden was quantitated in the placentas of wildtype and Trem2^-/-^ mice. Our data showed that the parasite burden was significantly higher in the placenta of Trem2^-/-^ mice than that of wildtype mice, demonstrating that deficiency of Trem2 facilitated *T*. *gondii* amplification (Fig 2D). *T*. *gondii*-induced placental inflammation is an important cause of adverse pregnancy [36], and given that Trem2 has a crucial role in regulating inflammation [15], we further asked whether Trem2 affects the inflammatory responses at the maternal-fetal interface. By measuring the mRNA levels of several key cytokines [18,37], we found that *T*. *gondii* infection upregulated IL-1β, IL-12, and IFN-γ expression in both wildtype and Trem2^-/-^ mice as compared to their corresponding uninfected controls (Fig 2E). However, there was no difference of IL-12 and IFN-γ levels between wildtype and Trem2^-/-^ mice after infection, indicating that Trem2 may not regulate the production of IFN-γ and IL-12 in the context of *T*. *gondii* infection. The expression of TNF-α, IL-10, TGF-β and G-CSF were comparable between infected and uninfected groups. In addition, Trem2^-/-^, but not wildtype mice exhibited higher IL-6 following infection. Notably, following *T*. *gondii* infection, Trem2^-/-^ mice expressed higher level of IL-1β as compared to wildtype mice (Fig 2E), indicating that Trem2 signaling may control the inflammatory responses in the placentas by modulating pro-inflammatory IL-1β expression.

It is reported that Trem2 deficiency augmented M1 polarization [38]. *T*. *gondii* infection in mice leads to adverse pregnancy outcomes via inducing M1 polarization of decidual macrophage, which alters the immunosuppressive micro-environment at the maternal-fetal interface [39]. To assess whether Trem2 deficiency affects decidual macrophage polarization in *T*. *gondii* infection, we analyzed transcript levels of M1-associated (CD86 and iNOS) and M2-associated markers (CD206 and Arg1) in the mouse placentas. Our results showed that in the context of *T*. *gondii* infection, CD86 and iNOS expression were increased in wildtype and Trem2^-/-^ mice, whereas CD206 and Arg1 expression were decreased, indicating an increased M1-type bias and a decreased M2-type bias. Moreover, infected Trem2^-/-^ mice showed a more pronounced M1-type bias compared to infected wildtype mice (S2A Fig). To further confirm the role of Trem2 on M1 polarization, we isolated BMDMs from wildtype and Trem2^-/-^ mice and stimulated them with *Tg*Ag *in vitro*. The analysis of flow cytometry showed that increased CD86 but decreased CD206 percentage were found in both *Tg*Ag-stimulated wildtype and Trem2^-/-^ BMDMs as compared with their control groups. *Tg*Ag-stimulated Trem2^-/-^ BMDMs showed a higher percentage of CD86, compared to *Tg*Ag-stimulated wildtype BMDMs (S2B and S2C Fig). Thus, our data suggested that Trem2 deficiency in mice may further contribute to the M1-type polarization of decidual macrophages induced by *T*. *gondii* infection.

### *T*. *gondii* infection causes down-regulation of Trem2/Syk/PI3K signaling in placental tissues

**Fig 3:**
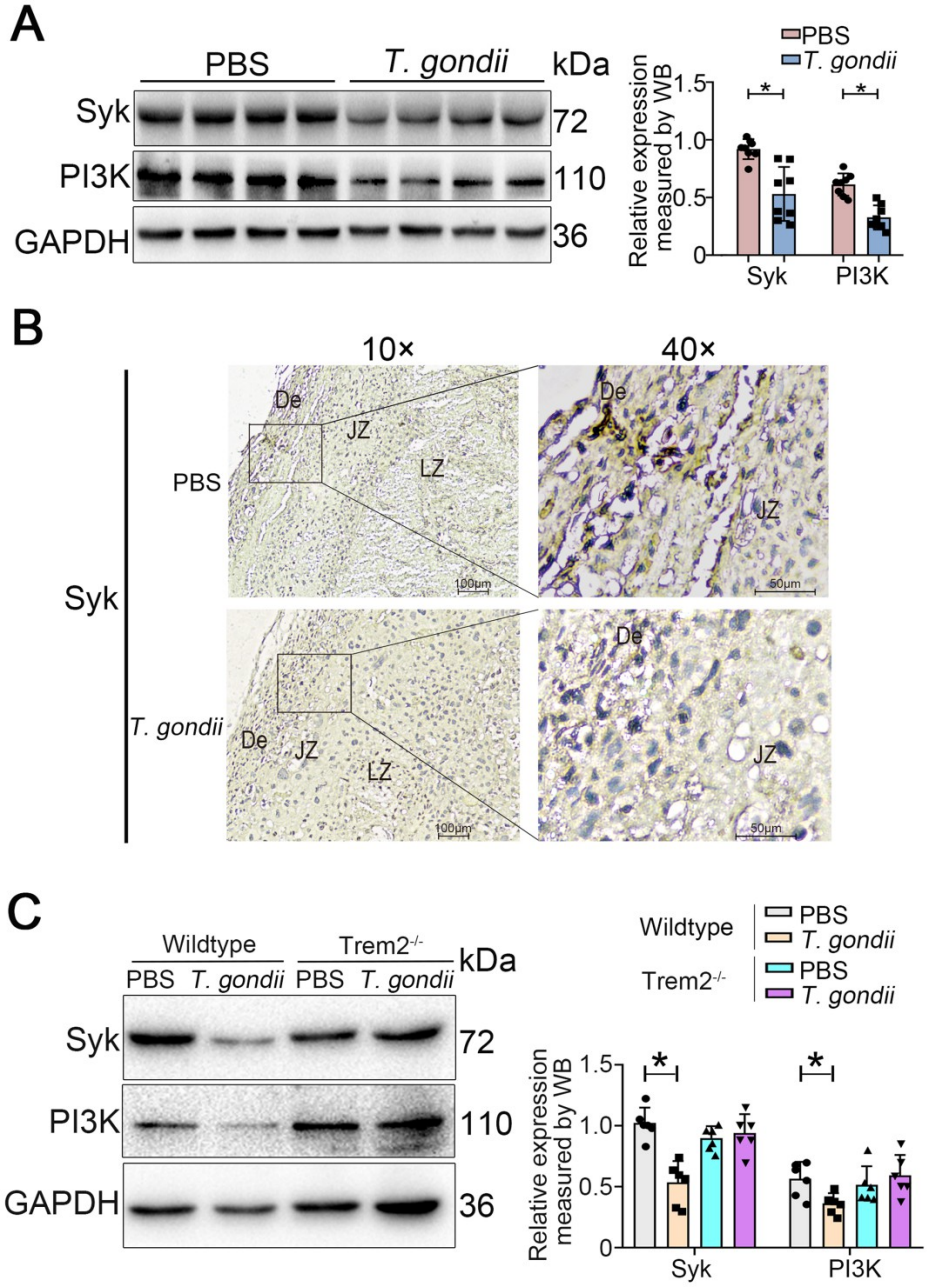
*T*. *gondii* infection inhibits the expressions of Syk and PI3K in mouse placentas. (A) Syk and PI3K protein levels in normal and infected mouse placentas were analyzed by immunoblot. Each data point represents the placenta of an individual pregnant mouse (n = 8 mice). (B) Immunohistochemistry of normal and infected mouse placentas, which were immunostained with anti-Syk antibody and counterstained with hematoxylin. (C) Syk and PI3K protein levels in wildtype and Trem2^-/-^ pregnant mouse placentas with or without *T*. *gondii* infection were analyzed by immunoblot. Data point represents the placenta of a single pregnant mouse (n = 6 mice). Data were presented as mean ± SD. Statistical analysis was conducted using one-way ANOVA with Tukey’s multiple comparisons (A and C). *: *P* < 0.05.

Syk, as a central node of the Trem2 signaling pathway, is greatly implicated in the regulation of macrophage function and has been a research hotspot in recent years [40–42]. In parallel, PI3K is also a core molecule in Trem2 signal transduction and can be recruited and activated by Syk [43]. We sought to explore whether the Trem2/Syk/PI3K signaling pathway is implicated in the mechanism of adverse pregnancy triggered by *T*. *gondii*. We hypothesized that *T*. *gondii* can modulate Trem2 signaling, leading to the inhibition of Syk/PI3K in the placentas. To test our hypothesis, protein expression of Syk and PI3K in the placenta samples was assessed by immunoblot. The result analysis manifested that *T*. *gondii* infection significantly inhibited the expression of Syk and PI3K in mouse placentas (Fig 3A). Immunohistochemistry results showed a similar down-regulation of Syk expression triggered by *T*. *gondii* (Fig 3B), indicating that Trem2 might be implicated in the pathological mechanism of *T*. *gondii* infection through its downstream Syk and PI3K signaling. To corroborate that Trem2 is required for Syk/PI3K signaling in the responses to *T*. *gondii*, we further examined changes in Syk and PI3K expression following Trem2 knockout using western blot analysis. Data analysis showed that no significant difference of Syk and PI3K expression was detected in the mouse placentas of Trem2^-/-^ groups with or without infection (Fig 3C). We concluded that Trem2 is required for the activation of Syk and PI3K signaling pathway during *T*. *gondii* infection.

To address the question whether *T*. *gondii* manipulates other pathways, we test several other important signaling molecules of Trem2 downstream (NFκB, mTOR and ERK), which are also essential signaling molecule of Trem2 downstream and involved in Trem2-related biological functions [15]. Our results showed that *T*. *gondii* infection down-regulated the protein expression of these molecules; however, no significant difference was found between wildtype and Trem2^-/-^ mice following infection, indicating that the regulation of these pathways is in a Trem2-independent manner (S3 Fig). In all, our data indicate that Trem2 is involved in *T*. *gondii*-induced adverse pregnancy by regulating Syk/PI3K signaling in decidual macrophages.

### The inhibition of the Trem2-Syk-PI3K axis characterizes macrophage responses to *T*. *gondii* antigens

**Fig 4:**
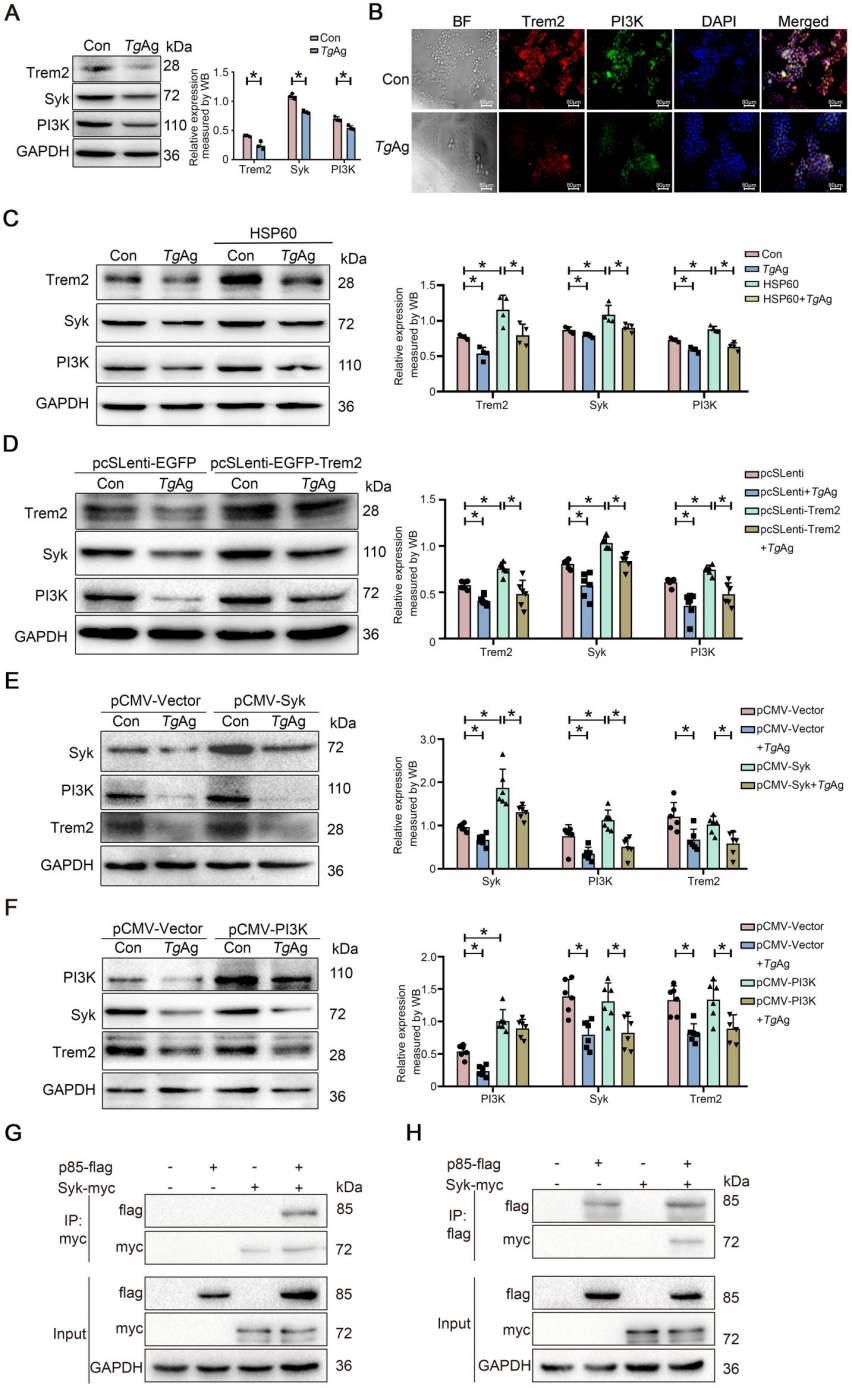
*Tg*Ag inhibits the Trem2-Syk-PI3K axis. (A) RAW264.7 cells were stimulated with solvent control and *Tg*Ag for 48 h respectively. The expressions of Trem2, Syk and PI3K proteins were detected by immunoblot, and the statistical analysis was conducted by Image J. Data represent the results of three independent experiments. (B) Immunofluorescence images of the co-localization Trem2 (red) and PI3K (green) in RAW264.7 cells stimulated with either control or *Tg*Ag were shown. (C) RAW264.7 cells were treated with different stimuli (control, *Tg*Ag, HSP60 and *Tg*Ag+HSP60) for 48 h. The expressions of Trem2, Syk, and PI3K were shown, as analyzed by immunoblot and quantification. Data represent the results of four independent experiments. (D) The lentiviral control vector and Trem2 lentiviral overexpression vector were transfected into RAW264.7 cells, respectively. After 12 h of overexpression, *Tg*Ag was added for stimulation for 48 h. The expressions of Trem2, Syk, and PI3K were measured by Immunoblot and quantified by Image J. Data represent the results of six independent experiments. (E) RAW264.7 cells were transfected with the vector plasmid and Syk recombinant plasmid, respectively. After 12 h of overexpression, *Tg*Ag was added for stimulation for 48 h. The expressions of Trem2, Syk, and PI3K were measured by Immunoblot and quantified by Image J. Data represent the results of six independent experiments. (F) RAW264.7 cells were transfected with the vector plasmid and PI3K recombinant plasmid, respectively. After 12 h of overexpression, *Tg*Ag were added for stimulation for 48 h. The expressions of Trem2, Syk, and PI3K were measured by Immunoblot and quantified by Image J. Data represent the results of six independent experiments. (G and H) HEK-293T cells were transfected separately or co-transfected with myc-tagged Syk and flag-tagged p85 for 48 h. Cell lysates were immunoprecipitated with myc antibody (G) or flag antibody (H), and the immunoblots with myc and flag antibodies are shown. Data were presented as mean ± SD. Statistical analysis was conducted using two-tailed unpaired Student’s *t*-test (A) or one-way ANOVA with Tukey’s multiple comparisons test (C, D, E and F). *: *P* < 0.05. *Tg*Ag: *T*. *gondii* antigens.

Given the complexity of Syk and PI3K pathways, we next sought to explore the independence and correlation of Syk and PI3K signaling in macrophage responses to *T*. *gondii*. We used mouse macrophage cell line (RAW264.7) to establish an *in vitro T*. *gondii* antigens (*Tg*Ag)-stimulated cell model. Consistent with the *in vivo* findings, *Tg*Ag stimulation significantly reduced the expressions of Syk, PI3K, and Trem2 in RAW264.7 cells (Fig 4A). Similarly, results of immunocytofluorescence demonstrated the decreased fluorescence intensity of Trem2 and PI3K-labeled proteins by *Tg*Ag stimulation (Fig 4B). Next, we sought to confirm that Syk and PI3K were downstream signals of Trem2 *in vitro* cell culture. To investigate this, we stimulated RAW264.7 cells with the Trem2 agonist HSP60 and found that HSP60 significantly increased the expression of Trem2, Syk, and PI3K; however, *Tg*Ag inhibited the elevated expression of Trem2, Syk, and PI3K induced by HSP60 (Fig 4C). We further transfected the Trem2 lentiviral overexpression vector into macrophages and corroborated that Trem2 overexpression can promote the expression of Syk and PI3K, while *Tg*Ag could inhibit the elevated expression of Trem2, Syk and PI3K induced by Trem2 overexpression (Fig 4D).

We also transfected Syk and PI3K overexpression plasmids into macrophages to test the regulation between Syk and PI3K. Western blot analysis indicated that increased expression of Syk could promote PI3K expression, while Trem2 expression remained unchanged (Fig 4E). In contrast, PI3K overexpression did not influence the expression of Trem2 and Syk (Fig 4F). Okkenhaug, K *et al* proposed that Syk can directly bind to the p85 subunit in PI3K, thereby recruiting and activating PI3K [44]. To validate this, we transfected Syk-3×Myc plasmid and p85-3×flag plasmid into 293T cells and confirmed the interaction between Syk and p85 through co-IP experiments (Fig 4G and 4H). Thus, we conclude that Trem2 signaling can activate Syk, which may subsequently recruit and activate PI3K through direct binding. Therefore, *T*. *gondii* may impair macrophage function via inhibiting the Trem2-Syk-PI3K axis.

### Trem2 modulates the migration, invasion, and proliferation of trophoblast cells

**Fig 5:**
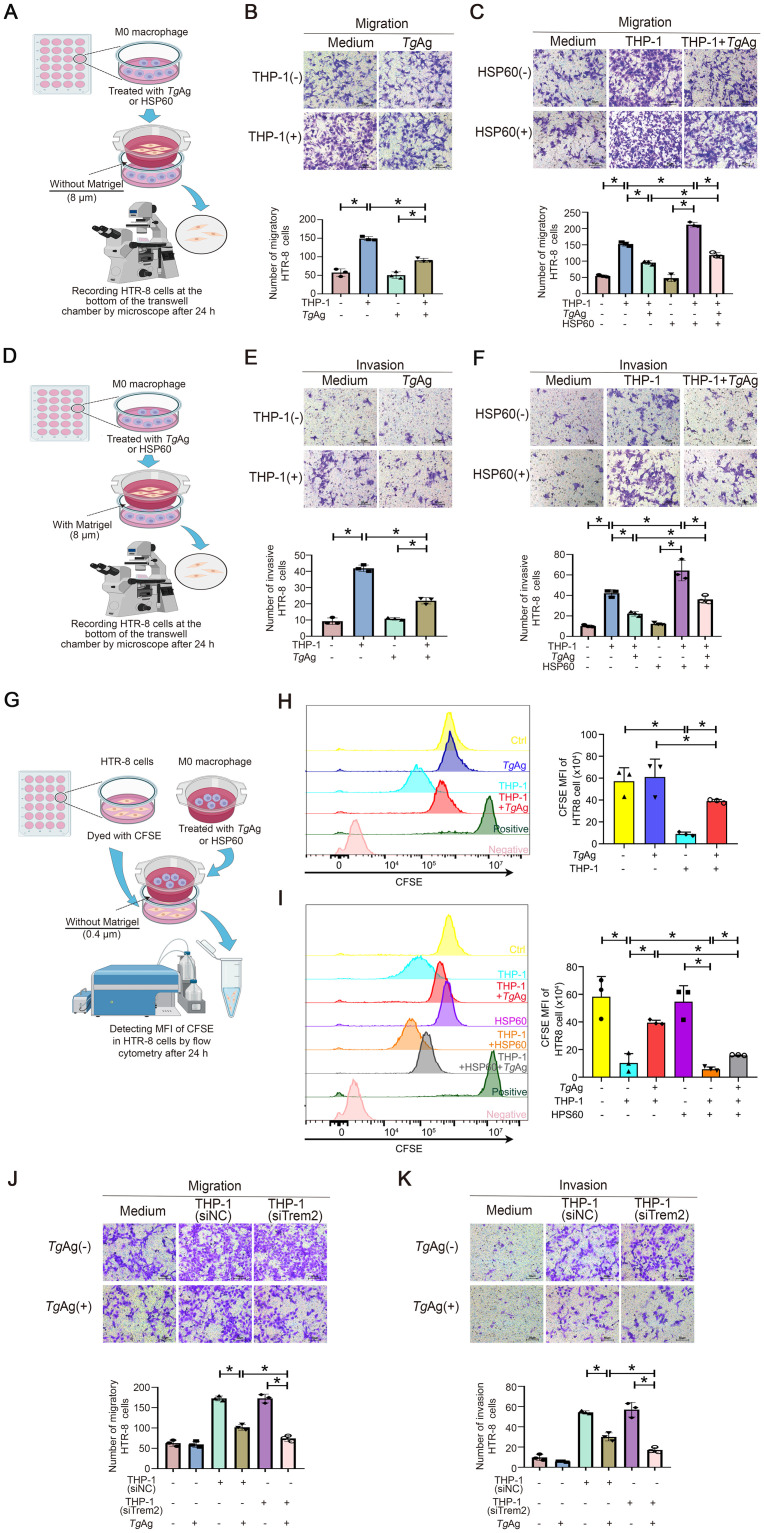
*Tg*Ag inhibits trophoblast cell migration, invasion, and proliferation via affecting macrophages. (A) Schematic diagram of migration assay of HTR-8 cells co-cultured with M0-type macrophages in a transwell system. (B) Migration assays for detection of the migratory capacity of HTR-8 cells co-cultured with control or M0-type macrophages with or without *Tg*Ag treatment for 24 h. Statistical analysis was conducted on the number of HTR-8 cells. Data represent the results of three independent experiments (n = 5 fields of view / group). (C) Migration assays for detection of the migratory capacity of HTR-8 cells co-cultured with control or *Tg*Ag-pretreated M0-type macrophages with or without HSP60 stimulation for 24 h. Statistical analysis was conducted on the number of HTR-8 cells. Data represent the results of three independent experiments (n = 5 fields of view / group). (D) Schematic diagram of invasion assay of HTR-8 cells co-cultured with M0-type macrophages in a transwell system. (E) Matrigel invasion assays for detection of the invasive capacity of HTR-8 cells co-cultured with control or M0-type macrophages with or without *Tg*Ag treatment for 24 h. Statistical analysis was conducted on the number of HTR-8 cells. Data represent the results of three independent experiments (n = 5 fields of view / group). (F) Matrigel invasion assays for detection of the invasive capacity of HTR-8 cells co-cultured with control or *Tg*Ag-pretreated M0-type macrophages with or without HSP60 stimulation for 24 h. Statistical analysis was conducted on the number of HTR-8 cells. Data represent the results of three independent experiments (n = 5 fields of view / group). (G) Schematic diagram of proliferation assay of HTR-8 cells co-cultured with M0-type macrophages in a transwell system. (H) Flow cytometry for the evaluation of proliferation of CFSE-dyed HTR-8 cells, co-cultured with control or M0-type macrophages with or without *Tg*Ag treatment for 24 h. Statistical analysis was conducted on the MFI of HTR-8 cells. Data represent the results of three independent experiments. (I) Flow cytometry for the evaluation of proliferation of CFSE-dyed HTR-8 cells, co-cultured with control or *Tg*Ag-pretreated M0-type macrophages with or without HSP60 stimulation for 24 h. Statistical analysis was conducted on the MFI of HTR-8 cells. Data represent the results of three independent experiments. (J) Migration assays for detection of the migratory capacity of HTR-8 cells co-cultured with control or *Tg*Ag-pretreated M0-type macrophages transfected with siRNA-NC (si-NC) or siRNAs against Trem2 (si-Trem2) for 24 h. Statistical analysis was conducted on the number of HTR-8 cells. Data represent the results of three independent experiments (n = 5 fields of view / group). (K) Matrigel invasion assays for detection of the invasive capacity of HTR-8 cells co-cultured with control or *Tg*Ag-pretreated M0-type macrophages transfected with si-NC or si-Trem2 for 24 h. Statistical analysis was conducted on the number of HTR-8 cells. Data represent the results of three independent experiments (n = 5 fields of view / group). Data were presented as mean ± SD. Statistical analysis was conducted using one-way ANOVA Tukey’s multiple comparisons test (B, C, E, F, H, I, J, and K). *: *P* < 0.05. *Tg*Ag: *T*. *gondii* antigens. The schematic diagram is created with Biorender.com.

Decidual macrophages interact with trophoblast cells and play a major role in maintaining their normal function [45,46]. To investigate whether *Tg*Ag affects the crosstalk between macrophages and trophoblast cells [47], we co-cultured HTR-8 cells and M0-type macrophages derived from THP-1 cells to imitate cell-cell interaction at the maternal-fetal interface (Fig 5A, 5D and 5G). We found that THP-1-differetiated macrophages can promote the migration and invasion of trophoblast cells; while *Tg*Ag treatment dampened this effect. Notably, *Tg*Ag failed to directly affect the migratory and invasive capacity of trophoblast cells (Fig 5B and 5E). When measuring the proliferation ability of trophoblast cells, we also found the similar phenomenon. In the presence of macrophages, the proliferation of trophoblast cells was significantly augmented, whereas *Tg*Ag stimulation in the co-culture significantly suppressed trophoblast proliferation. In addition, *Tg*Ag had no impact on the proliferation of trophoblast cells in the absence of macrophages (Fig 5H). Hence, our results suggested that *Tg*Ag might inhibit the functions of trophoblast cells only by acting on macrophages as an "intermediate bridge".

Given that *Tg*Ag may affect macrophage function via down-regulating Trem2 signaling, we attempted to rescue Trem2 activation in *Tg*Ag-stimulated macrophages via HSP60. The results corroborated that HSP60 can foster the migration, invasion, and proliferation of trophoblast cells via acting on macrophages. Likewise, the addition of HSP60 to *Tg*Ag-treated macrophages can significantly improve the inhibited biological functions of trophoblast cells (Fig 5C, 5F and 5I). To further assess the role of Trem2 in macrophage-trophoblast crosstalk, we knocked down Trem2 in THP-1 cells by siRNA-Trem2 and co-cultured them with HTR-8 cells, followed by the detection of trophoblast migration and invasion (S4 Fig). Our data showed that Trem2 knockdown in THP1 cells further promoted the inhibitory effect of *Tg*Ag on HTR-8 migration and invasion (Fig 5J and 5K).

**Fig 6:**
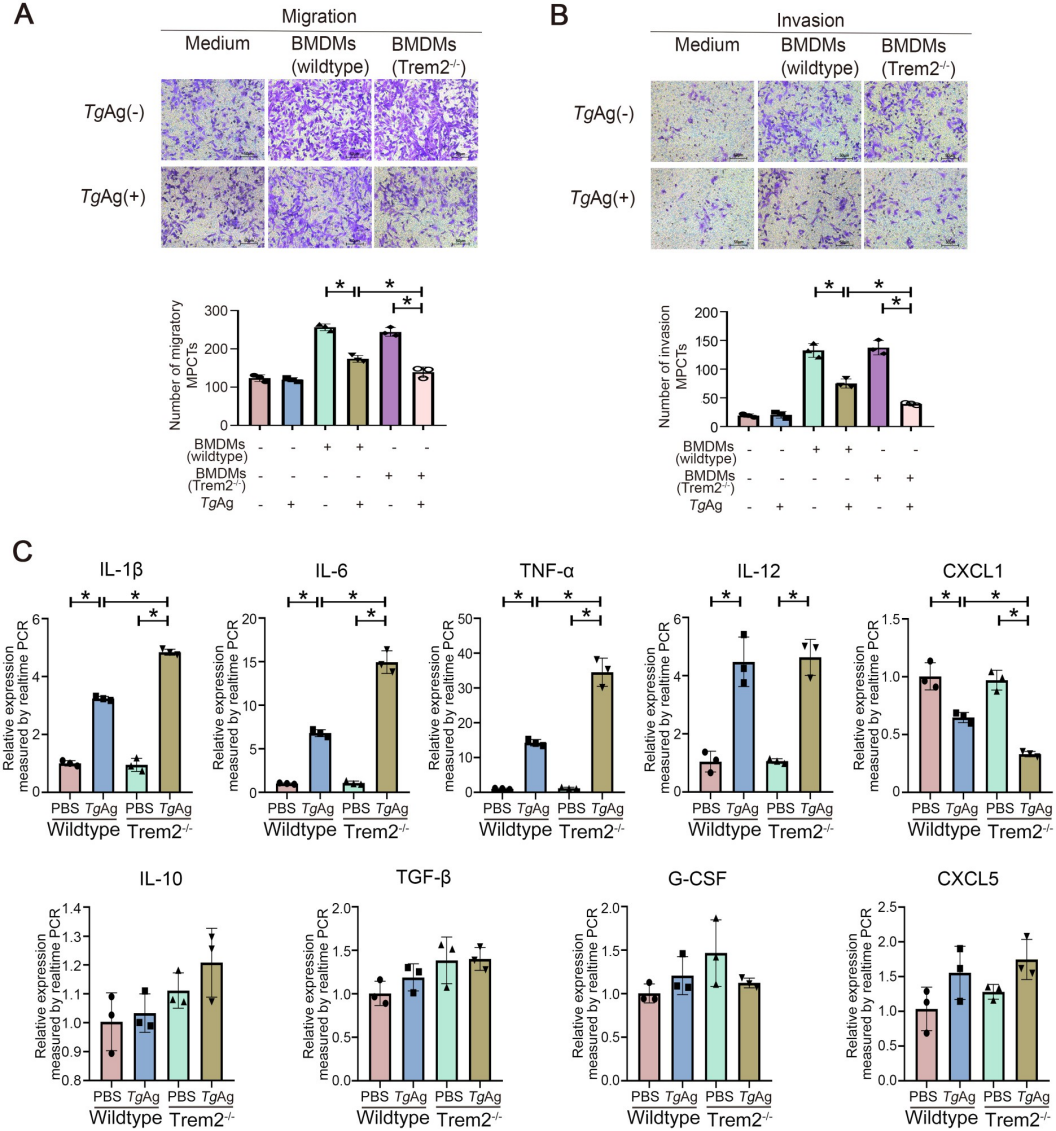
Trem2 deficiency in BMDMs promotes inflammatory factor production and inhibits CXCL1. (A) Migration assays for detection of the migratory capacity of MPCTs co-cultured with control or *Tg*Ag-pretreated BMDMs from wildtype mice and Trem2^-/-^ mice. Statistical analysis was conducted on the number of MPCTs. Data represent the results of three independent experiments (n = 5 fields of view / group). (B) Matrigel invasion assays for detection of the invasive capacity of MPCTs co-cultured with control or *Tg*Ag-pretreated BMDMs from wildtype mice and Trem2^-/-^ mice. Statistical analysis was conducted on the number of MPCTs. Data represent the results of three independent experiments (n = 5 fields of view / group). (C) mRNA levels of IL-1β, IL-6, IL-10, IL-12, TNF-α, TGF-β, G-CSF, CXCL1 and CXCL5 in the BMDMs were assayed by real-time PCR. Data represent the results of three independent experiments. Data were presented as mean ± SD. Statistical analysis was conducted using one-way ANOVA with Tukey’s multiple comparisons test (A, B and C). *: *P* < 0.05. *Tg*Ag: *T*. *gondii* antigens.

To further confirm that Trem2 is involved in the *Tg*Ag-mediated inhibition of macrophage-trophoblast crosstalk, we also generated bone marrow-derived macrophages (BMDMs) from Trem2^-/-^ mice, and co-cultured them with mouse placental chorionic trophoblast cells (MPCTs), followed by the assay of migration and invasion of MPCTs (Fig 6A and 6B). Consistently, we found that macrophages can promote the migration and invasion of trophoblast cells, whereas *Tg*Ag failed to directly affect behaviors of trophoblast cells. However, *Tg*Ag may inhibit the function of trophoblast cells by modulating macrophages. We further found that the deficiency of Trem2 in macrophages heightened the inhibitory effect of *Tg*Ag on the migration and invasion of trophoblast cells. There results confirmed that Trem2 deficiency can aggravate the impairment of *Tg*Ag on this crosstalk.

Decidual macrophages can regulate the biological functions of trophoblast cells via secreting inflammatory cytokines and chemokines. In particular, the inflammatory responses induced by macrophages impairs capabilities of trophoblast cells [8,47]. To explore the mechanism by which *Tg*Ag affects macrophage-trophoblast crosstalk, we assessed the mRNA levels of IL-1β, IL-6, IL-10, IL-12, TNF-α, TGF-β, G-CSF, CXCL1, and CXCL5 in BMDMs in the above co-culture system by real-time PCR. We found that *Tg*Ag treatment increased pro-inflammatory factors (IL-1β, IL-6, IL-12 and TNF-α) and decreased chemokine CXCL1 in wildtype BMDMs. Further analysis showed that Trem2 deficiency resulted in higher pro-inflammatory factors (IL-1β, IL-6 and TNF-α) but a lower chemokine (CXCL1) in BMDMs (Fig 6C). These results demonstrated that *Tg*Ag may trigger dysregulated responses in decidual macrophages, leading to impaired trophoblast cell functions and adverse pregnancy outcomes in *T*. *gondii* infection.

## Discussion

Our study identified the transmembrane receptor Trem2 as a pivotal driver for decidual macrophages in the responses to *T*. *gondii* invasion during pregnancy in mice. By constructing wildtype and Trem2^-/-^ pregnant mouse models, we explored the repercussion of Trem2 on pregnancy outcomes triggered by *T*. *gondii*. Experimental data manifested that *T*. *gondii* infection could lead to a significant increase in the proportion of decidual macrophages in wildtype mice, but Trem2 expression on the surface of decidual macrophages was observably reduced. In addition, the proportion of decidual macrophages in Trem2^-/-^ mice was obviously declined after *T*. *gondii* infection, which was opposite to the changes in wildtype mice. The reason for the increase of decidual macrophages may be that *T*. *gondii* invasion in mouse placenta can trigger an innate immune response, resulting in the recruitment of a large number of immune cells, including macrophages, to the placentas [48]. Nevertheless, compared to the predominance of M2-type decidual macrophages at the maternal-fetal interface, *T*. *gondii* infection can foster a severe immune-inflammatory response, which mainly induces polarization of decidual macrophages towards the M1-type [9,39,49]. We also demonstrated that Trem2 deficiency can further promote M1-type polarization of decidual macrophages in *T*. *gondii*-infected mice, and given that M1-type macrophages can induce adverse pregnancy, augmented M1-type bias in Trem2^-/—^infected mice may be a cause of exacerbated adverse pregnancy. Additionally, Trem2 has been shown to not merely facilitate macrophage recruitment to pathological sites [21,34], but also enhance macrophage survival in inflammatory and infectious states [15,50]. Due to the lack of Trem2 signal transduction, Trem2^-/-^ macrophages exhibit excessive autophagy, pyroptosis and apoptosis, accompanied by impaired anabolism and energy metabolism in neurodegenerative or infectious diseases, resulting in the suppression of macrophage survival, proliferation and aggregation [34,51,52]. Our study showed that Trem2 deficiency in *T*. *gondii*-infected mice led to a decrease in the proportion and number of decidual macrophage. Therefore, Trem2 signaling may promote macrophage recruitment as well as survival in wildtype mice infected with *T*. *gondii*, whereas the lack of Trem2 may induce damaged or death of decidual macrophages in *T*. *gondii* infection, leading to a reduction in their quantity.

Trem2 has been extensively researched primarily on the capacity of microglia, which foster microglial responses to neurodegenerative diseases like Alzheimer’s disease [53]. While Trem2 has also been shown to be physiologically expressed on decidual macrophages [26], it remains unknown whether Trem2 drives decidual macrophages to maintain normal pregnancy and resist pathogen invasion. An earlier analysis of decidual macrophage gene expression profiles showed that Trem2 gene was significantly upregulated in decidual macrophages compared with blood macrophages during pregnancy [25]. A recent single-cell transcriptomic study also found a decrease in Trem2^+^ macrophages in PE placentas [26]. Trem2 signaling can not only positively regulate macrophage phagocytosis, thereby promoting the phagocytosis of apoptotic cells, but also negatively regulate the inflammatory responses [54,55]. This special dual function of Trem2 is contributory to the formation of immune tolerance mechanism at the maternal-fetal interface, facilitating the clearance of apoptotic cells and the regulation of immune responses during pregnancy. Therefore, these studies indicate that Trem2 signaling might be a major pathway by which decidual macrophages are implicated in the maintenance of normal pregnancy. In the present study, we compared the pregnancy outcomes of wildtype and Trem2^-/-^ mice. Fetal size and weight in Trem2^-/-^ mice were found to have no significant difference, nor was there a difference in the proportion of decidual macrophages from wildtype mice. We speculate that decidual macrophages do not simply ignore the role of Trem2 during normal pregnancy in Trem2^-/-^ mice, but are forced into a state of stress in which the impaired function due to Trem2 deficiency may be compensated for by other signals (e.g., vascular endothelial growth factor (VEGF), programmed cell death-1 (PD-1)/PD-1 ligand -1 (PD-L1), and tumor necrosis factor α) to maintain a homeostasis [56,57]. Future study is warranted to explore the role and mechanisms of Trem2 in maintaining normal pregnancy.

It is highly significant that we detected a down-regulation of Trem2 expression in decidual macrophages of wildtype pregnant mice after *T*. *gondii* infection. The infected Trem2^-/-^ mice exhibited even more severe fetal developmental restriction and fetal death as compared with the infected wildtype pregnant mice. It has been reported that Trem2^-/-^ mice are more susceptible to pathogen infection. For example, macrophage-mediated killing assay showed that Trem2 deficiency could reduce the number of macrophages by inducing pyroptosis, thereby resulting in the reduced clearance of pyogenic bacteria [51]. Moreover, the Trem2 gene sequence correlates with certain parasite resistance genes. An increased parasite expansion was reported in a Trem2^-/-^ mouse model of *Plasmodium berghei* infection [35]. Our data showed that the *T*. *gondii* burden was significantly higher in the placenta of Trem2^-/-^ mice than that of wildtype mice, demonstrating that deficiency of Trem2 facilitated *T*. *gondii* amplification. Trem2, as a transmembrane innate immune receptor, promotes macrophage survival [38] and functions (e.g., phagocytosis [58], autophagy [59] or lysosomal activity [60]). In addition, Trem2 deficiency caused a decrease in the number of decidual macrophages in *T*. *gondii*-infected mice. Therefore, dysregulated decidual macrophages due to Trem2 deficiency may lead to a failure to control parasites.

However, it is worth noting that Trem2 may promote immune evasion of pathogens, via negatively regulating inflammatory responses [18,50]. IL-12 and IFN-γ, which are mainly produced by dendritic cells and lymphocytes respectively, are critical for the killing of *T*. *gondii* [61]. We analyzed the transcript levels of IFN-γ and IL-12 in placental tissues and found that *T*. *gondii* infection resulted in increased IFN-γ and IL-12 in both wildtype and Trem2^-/-^ mice as compared with their uninfected control mice. However, there is no significant difference of these cytokines between infected wildtype and Trem2^-/-^ mice. This result suggests that Trem2, which is mainly expressed on macrophages, may not regulate the production of IFN-γ and IL-12 in the context of *T*. *gondii* infection. In addition, it is reported that Trem2 activation inhibited IL-1β secretion, as evidenced by reduced IL-1β secretion in Trem2-overexpressing macrophages upon *E*. *coli* challenge [62]. Consistently, our study showed the elevated IL-1β in the placentas of Trem2^-/-^ mice compared to wildtype mice following infection. Importantly, although lacking IL-1β in mice has no influence on *T*. *gondii* control and immune cell infiltration [63], increased IL-1β in placental tissue might be linked to an imbalanced immune system and a procoagulant state that accounts for pregnancy loss [64]. Thus, uncontrolled *T*. *gondii* amplification and excessive inflammatory responses might be the reason for the exacerbated adverse pregnancy outcomes in Trem2^-/-^ mice.

Furthermore, consistent with the mouse model data, the *in vitro* co-culture results provide a new insight into the pathology of *T*. *gondii*-induced adverse pregnancy outcomes. We demonstrated that *Tg*Ag can affect macrophage-trophoblast crosstalk and thereby inhibit the proliferation, migration, and invasion of trophoblast cells. Here, by using an *in vitro* co-culture model, we report that macrophages can promote biological functions of trophoblast cells, including the proliferation, migration, and invasion. In contrast, *Tg*Ag stimulation can inhibit the effect of macrophages on capacity of trophoblast cells, suggesting that *T*. *gondii* may inhibit the physiological functions of trophoblast cells by affecting macrophages. More intriguingly, we found that HSP60 improved the inhibitory effect of *Tg*Ag on Trem2 expression in macrophages. This unveils that Trem2 is a key target mediating the crosstalk between macrophages and trophoblast cells during *T*. *gondii*-damaged pregnancy. Decidual macrophages regulate the biological functions of trophoblast cells by secreting cytokines, and Trem2 is a crucial signaling molecule that regulates macrophage cytokines [8,54]. In our experiments, we found that Trem2 deficiency in BMDMs promoted the inhibition of trophoblast cell biological functions by *Tg*Ag. Meanwhile, *Tg*Ag treatment induced the transcription of inflammatory factors (IL-1β, IL-6 and TNF-α) in BMDMs, which was further promoted by Trem2 deficiency. A previous study showed that, in co-culture experiments, M1-type macrophages inhibited the migration and invasion of HTR-8 cells via releasing TNF-a, and an anti-TNF-α antibody reversed this effect [65]. Hence, Trem2 deficiency in BMDMs exacerbated the inhibition effect of *Tg*Ag on trophoblast cell biological functions by excessive inflammatory responses.

It has been shown that *T*. *gondii* can secrete virulence factors that affect macrophage cytokine secretion, with CXCL1 secretion being significantly lower in the infected macrophages than in the uninfected macrophages [66]. Additionally, inhibition of Syk signaling was shown to suppress the expression of CXCL1, which can be secreted by macrophages to promote trophoblast migration [8,67]. Consistently, we demonstrated that mRNA levels of CXCL1 were significantly reduced in *Tg*Ag-treated BMDMs. We speculate that *Tg*Ag may inhibit CXCL1 secretion or other related chemokine secretion by downregulating Trem2/Syk/PI3K signaling in macrophages, thereby inhibiting the migratory capacity of trophoblast cells. Thus, *Tg*Ag may inhibit the biological function of trophoblast cells by inducing pro-inflammatory responses and inhibiting CXCL1 secretion in macrophages [8], in which Trem2 signaling plays a favorable regulatory role. We also explored the molecular mechanisms by which decidual macrophages respond to *T*. *gondii* infection. We demonstrated that *T*. *gondii* infection can inhibit the Trem2-Syk-PI3K signaling pathway. Some research has demonstrated that Trem2-dependent phagocytosis relies on the activation of Syk and PI3K signaling, and that Trem2 regulates inflammatory responses by regulating Syk and PI3K signaling [68–70]. Additionally, Syk and PI3K signaling in macrophages was found to regulate the expression of cytokines (e.g., IL-1β, IL-6, TNF-α and CXCL1) [67,71]. Accordingly, along these lines, our data suggest that Trem2 may regulate phagocytosis and inflammatory responses to *T*. *gondii* through the downstream Syk/PI3K signaling pathway, while *T*. *gondii* impairs macrophage function required for maintaining the migration, invasion, and proliferation of trophoblast cells by inhibiting the Trem2-Syk-PI3K signaling pathway.

**Fig 7:**
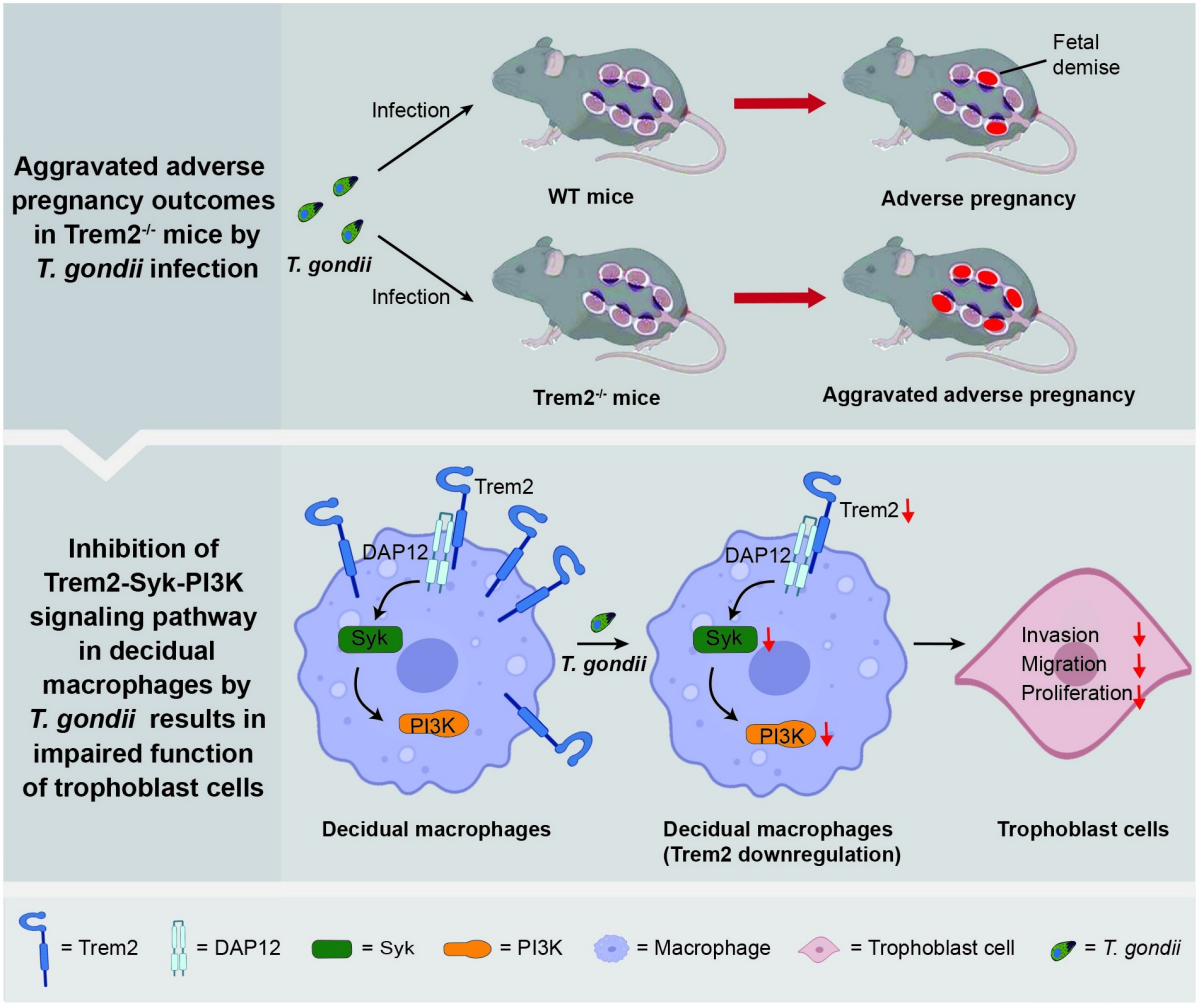
Schematic diagram of Trem2’s impact on decidual macrophages in *T*. *gondii*-induced adverse pregnancy outcomes. *T*. *gondii* infection results in adverse pregnancy outcomes in wildtype mice, paralleled by the down-regulation of Trem2 on the surface of decidual macrophages. Under normal pregnancy conditions, Trem2 signaling becomes activated and initiates the recruitment of downstream Syk and PI3K signaling, thereby activating the Trem2-Syk-PI3K pathway. However, *T*. *gondii* infection inhibits Trem2-Syk-PI3K pathway in decidual macrophages, leading to the impaired migration, invasion and proliferation of trophoblast cells. Furthermore, deficiency of Trem2 aggravates adverse pregnancy outcomes induced by *T*. *gondii* infection. The schematic diagram is created with Biorender.com.

In summary, we demonstrated that *T*. *gondii* infection could affect the quantity of decidual macrophages and Trem2 expression on its surface. Likewise, it impairs the migration, invasion, and proliferation of trophoblast cells through macrophages as an intermediate bridge, ultimately resulting in adverse pregnancy outcomes. As a paramount target, Trem2 deficiency aggravates adverse pregnancy outcomes induced by *T*. *gondii*, while Trem2 upregulation can improve the suppression of the physiological functions of trophoblast cells induced by *Tg*Ag. In parallel, we demonstrated that the Trem2-Syk-PI3K signaling pathway was involved in the pathological mechanism of adverse pregnancy induced by *T*. *gondii* (Fig 7). In conclusion, our study highlights Trem2 as a critical receptor of decidual macrophages that regulate *T*. *gondii* infection at the maternal-fetal interface. Our study provides a new perspective as well as a promising therapeutic avenue for adverse pregnancy outcomes triggered by *T*. *gondii* infection.

### Limitations of the study

Firstly, our studies on the role of decidual macrophages on *T*. *gondii*-induced adverse pregnancy outcomes were limited to Trem2^-/-^ mouse models. As there are no surface markers that can be specifically targeted to the mouse placentas, we have not yet constructed a placenta-specific Trem2^-/-^ mouse model. Secondly, although our study reveals the role of Trem2 as a mediator of the cross talk between macrophages and trophoblast cells induced by *Tg*Ag, we do not have evidence as to whether or how *Tg*Ag binds to Trem2. Further study is warranted for the underlying mechanism of parasite protein and host cell interaction.

## Materials and methods

### Ethics statement

All experiments in mice were performed in strict accordance with the Guidelines for the Management and Use of Laboratory Animals (Science Press of China, 2016). The experimental manipulation in this study was approved by the Animal Care and Use Committee of Nantong University (No. P20230302-013).

### Mice and quantification of *T*. *gondii* burden

6-to 8-week-old ICR and C57BL/6 mice were ordered from Nantong University Laboratory Animal Centre, while B6/JGpt-Trem2 em1Cd3332in1/Gpt knockout (Trem2^-/-^) mice on the B6 background were constructed by Gempharmatech Co., Ltd (Nanjing, Jiangsu, China) based on CRISPR/Cas9 technique. *T*. *gondii* tachyzoites were gifted by Prof. Yong Wang from Nanjing Medical University and passaged in our laboratory by intraperitoneal injection into ICR mice. Studies on mouse pregnancy were timed by the presence of vaginal plug, defined as G0.5 [72]. Both wildtype and Trem2^-/-^ pregnancy mice were i.p. injected with 300 tachyzoites at G8.5 and euthanized via CO_2_ asphyxiation at G17.5. All mouse placentas were collected, and fetuses were photographed and weighed. Fetal development was assayed by fetal weight and fetal size, which was measured by occipito-frontal diameter (OF) multiplied by crown-rump length (CRL). The *T*. *gondii* burden was quantitated in the placentas of wildtype and Trem2^-/-^ mice as described previously [73].

### Cell culture and treatment

*Tg*Ag was provided by the Department of Pathogen Biology, Nantong University [72]. All cell lines were ordered from the Cell Resource Center of Shanghai Institute of Life Science (Shanghai, China). The mouse monocytic cell line (RAW264.7) cells and human embryonic kidney 293T (HEK-293T) cells were routinely maintained in Dulbecco’s modified Eagle’s medium (DMEM; 2522444, Thermo Fisher Scientific, Massachusetts, USA), containing 10% fetal bovine serum (FBS; FSP500, ExCell Bio, Jiangsu, China) and 1% of antibiotic solution (100 U/mL penicillin and 100 μg/mL streptomycin). The HTR-8/SVneo trophoblast cell line (HTR-8) cells were maintained in RPMI-1640 medium containing 10% FBS, while the human monocytic cell line (THP-1) cells were cultured in RPMI-1640 medium (MA0315, MeilunBio, Liaoning, China) containing 0.05 mM β-Mercaptoethanol (M3148, MedcheMexpress, New Jersey, USA) and 10% FBS. M0-type macrophage was obtained via stimulating THP-1 cell with 100 ng/mL phorbol 12-myristate 13-acetate (PMA; HY-18739, MedcheMexpress) for 12 h. The RAW264.7 cells were treated with *Tg*Ag (5 μg/mL) or Trem2 agonist HSP60 (0.5 μg/mL) [74] for 48 h. MPCTs (PRI-MOU-00133) were procured from Zhong Qiao Xin Zhou Biotechnology (Shanghai, China), incubated in mouse placental trophoblast complete culture medium (PCM-M-133, Zhong Qiao Xin Zhou Biotechnology). BMDMs were prepared in our laboratory by extracting bone marrow cell suspensions from the tibia and femur of C57BL/6 wildtype mice and Trem2^-/-^ mice, and the bone marrow cells were cultivated and differentiated to macrophages for 7 days in DMEM containing 10% FBS and 40 ng/mL macrophage colony-stimulating factor (M-CSF; 315–02, Thermo Fisher Scientific). Cell culture was performed in a humidified incubator with 5% CO_2_ at 37 °C.

### Antibodies and reagents

Immunofluorescence, Western blot, immunohistochemistry, Co-immunoprecipitation (Co-IP), and flow cytometry were performed with the following antibodies. Anti-Syk antibody (13198), anti-mTOR antibody (2983), anti-CD11b antibody (93169) and anti-F4/80 antibody (30325) were ordered from Cell Signaling Technology (CST, Danvers, MA, USA). Anti-ERK1/2 antibody (ab184699) and anti-PI3K p110δ antibody (ab109006) were ordered from abcam (Cambridge, UK). Anti-NFκB antibody (sc-372), anti-flag antibody (sc-166384), anti-myc antibody (sc-40), and anti-sheep secondary antibody (sc2020) were purchased from Santa Cruz Biotechnology (CA, USA). Anti-GAPDH antibody (60004-1-1G) and anti-mouse secondary antibody (SA00001) were purchased from Proteintech (Chicago, USA), and anti-rabbit secondary antibody (BL003A) was purchased from Biosharp (Anhui, China). PE-conjugated antibody against mouse F4/80 (02922–60) was purchased from Biogems (New Jersey, USA). Anti-mouse Trem2 antibody (AF1729) was purchased from R&D Systems (Minnesota, USA). APC-conjugated antibody against mouse CD11b (17-0112-81/82), CD16/CD32 monoclonal antibody (14-0161-81), Rat IgG2b kappa APC-conjugated isotype control (17-4031-81), and Rat IgG2a kappa PE-conjugated Isotype control (12-4321-80) were ordered from eBioscience (San Diego, California, USA). BV421-conjugated antibody against mouse CD86 (564198) was obtained from BD Biosciences (New Jersey, USA). PE-Cy7-conjugated antibody against mouse CD206 (MR6F3), FITC-conjugated antibody against mouse Trem2 (MA5-28223), and Rat IgG1 kappa FITC-conjugated Isotype Control (11-4301-81) were ordered from Thermo Fisher Scientific. FITC-conjugated antibody against mouse CD3e (553061), PE-Cy7-conjugated antibody against mouse NK-1.1 (552878), APC-Cy7-conjugated antibody against mouse CD45 (557659), FITC IgG1 κ Isotype Control (553971), PE-Cy7 IgG2a κ Isotype Control, and APC-Cy7 IgG2b κ Isotype Control (552773) were obtained from BD Biosciences (New Jersey, USA).

### Plasmids, siRNA and transfection

The empty plasmids and the overexpression recombinant plasmids including pCMV-Pik3cd-Neo, pCMV-PIK3R1-3×FLAG-SV40-Neo, and pCMV-Syk-3×Myc-Neo were purchased from MiaoLing Biotech (Hubei, China). All the recombinant plasmids were verified by DNA sequence comparison. According to the reagent instructions, the plasmids were transfected into RAW264.7 or HEK-293T cells using the transfection reagent jetPRIME (101000046, Polyplus, Strasbourg, France), respectively. Trem2 overexpression lentiviral vector (pcSLenti-EF1-EGFP-P2A-Puro-CMV-Trem2-3×FLAG-WPRE) and its control were purchased from Obio Technology (Shanghai, China), and were transfected into RAW264.7 cells in accordance with the infection instructions. All small interference RNAs (siRNAs) against Trem2 and siRNA-negative control (NC) were designed and synthesized by Obio Technology, and the sequences for siRNAs were listed in S1 Table. The PMA-treated THP1 cells were transfected with the indicated siRNAs using INTERFERin (101000028, Polyplus) according to the manufacturer’s instructions.

### Real-time PCR analyses

Total RNA was extracted from cells or tissues using Trizol (15596–026, Invitrogen, California, USA). After cDNA synthesis using the RevertAid First Strand cDNA Synthesis Kit (K1621, Thermo Fisher Scientific), Real-time PCR analyses were performed using the TB Green Premix Ex Taq II Kit (RR420A, TAKARA, Osaka, Japan). The primer sequences for Real-time PCR were listed in S2 Table. The reference gene was GAPDH and relative gene level was assayed by the 2^-ΔΔCt^ method.

### Protein extraction and Western blot

All cell and tissue samples were lysed with RIPA lysis buffer containing phenylmethylsulfonyl fluoride (PMSF) for 30 min on ice for total proteins extraction. Protein samples were subjected to 4% - 20% sodium dodecyl sulfate-polyacrylamide gel electrophoresis (SDS-PAGE) and then semi-dry transferred to polyvinylidene fluoride (PVDF) membranes, which is followed by the blockage of 5% non-fat dried milk in TBST for 2 h at room temperature (RT). After blocking, PVDF membranes were washed and incubated with the corresponding primary antibodies overnight at 4 °C, followed by washing with TBST and incubating with secondary antibodies for 1 h at RT. Target proteins were visualized by an enhanced chemiluminescence (ECL) substrate (MA0186-1, Meilunbio) inside Bio-Rad ChemiDoc MP imaging system and analyzed using Image J software.

### Hematoxylin-Eosin (H&E) staining

Placenta of mice at G17.5 was fixed in neutral buffered formalin (10%) at RT and then embedded in paraffin. The embedded placenta sample was sectioned and then stained by hematoxylin and eosin. Photographs were then taken using a Leica DM5000B microscope (Leica, Vizsla, Germany) and the fields of view were randomly selected to observe the placenta morphology.

### Immunofluorescence staining and immunohistochemical staining

For immunohistochemistry, placenta sections were deparaffinized with xylene, followed by antigen retrieval with citric acid retrieval solution. After the blockage of endogenous peroxidase with 3% hydrogen peroxide, placenta sections were blocked with BSA (5%) and then incubated with the appropriate primary antibodies for 1 h at 37 °C. All sections were washed and incubated with the corresponding secondary antibody for 30 min at RT, followed by nuclear staining with hematoxylin. The samples were then sealed with neutral gum and dried before being observed and photographed with a microscope. For immunofluorescence staining, samples were incubated with the indicated primary antibodies overnight at 4 °C. All samples were then incubated with the corresponding secondary antibody for 1 h at RT away from light, followed by nuclear staining with DAPI. The film was then sealed with an anti-quencher, dried at RT with the avoidance of light, observed and photographed under a microscope.

### Flow cytometry

Mononuclear cells were isolated from mouse placenta, stained and analyzed using flow cytometry according to our previous report [75]. In brief, the mononuclear cells were incubated with Fc blocking (CD16/32 monoclonal antibody) prior to staining. The following fluorochrome-conjugated mAbs were utilized in the study: FITC-conjugated antibody against human/mouse Trem2, APC-conjugated antibody against mouse CD11b, PE-conjugated antibody against mouse F4/80, APC-Cy7-conjugated antibody against mouse CD45, FITC-conjugated antibody against mouse CD3e, PE-Cy7-conjugated antibody against mouse NK-1.1 and the corresponding isotype control antibody. Data analysis was performed using the flow cytometer (CytoFlex, California, USA) and FlowJo software.

### Transwell assay

The migration and invasion capacity of trophoblast cells was measured by Transwell (3422, Corning, New York, USA) assay. For trophoblast cell migration, 1 x 10^5^ macrophages (PMA-treated THP1 cells or BMDMs) were seeded into the lower chamber, while 3 x 10^4^ trophoblast cells (HTR-8 cells or MPCTs) were seeded into the upper chamber (8 μm). For trophoblast cell invasion, matrigel gel (356234, Corning) was applied to the upper chamber (8 μm), solidified and hydrated. Cells were respectively seeded into the upper and lower chambers (ibid.). After being co-cultured for 24 h, HTR-8 cells were blocked with paraformaldehyde, stained with crystal violet, photographed and counted under the microscope.

### Proliferation assay

HTR-8 cells were labeled with carboxyfluorescein succinimidyl ester (CFSE; C34570, Thermo Fisher Scientific) for 20 min at 37 °C in accordance with the manufacturer’s instructions. The fluorescently labeled-HTR-8 cells were then resuspended in RPMI-1640 medium containing 10% FBS and seeded into a six-well plate. Next, M0-type macrophages were treated with *Tg*Ag (5 μg/mL) or HSP60 (0.5 μg/mL), and subsequently added to the Transwell chamber (0.4 μm). After co-culturing for 24 h, HTR-8 cells were collected for flow cytometric analysis. The proliferation ability of HTR-8 cells was analyzed according to the mean fluorescence intensity (MFI) of CFSE.

### Co-immunoprecipitation

Cell was lysed with RIPA lysis buffer, supplemented with PMSF. According to the reagent instructions, primary antibody (2 μg) was added to the cell supernatant and incubated at 4 °C for 2 h. After the incubation, 20 μL of Protein A/G Plus-Agarose (SC-2003, Santa Cruz Biotechnology) was added and rotated at 4 °C overnight. The precipitate was harvested after centrifugation and washed with lysate. SDS-PAGE was then performed on the precipitate and input groups to detect the associated proteins.

### Statistical analysis

All data were presented as mean ± SD as indicated in the figure legends. All data were derived from three or more independent experiments. Statistical analysis was conducted using GraphPad Prism 8.0 software, with two-tailed unpaired Student’s *t*-test analysis between two groups or one-way analysis of variance (ANOVA) with Tukey’s multiple comparisons test between multiple groups. *P* < 0.05 was considered significant difference.

## Supporting information

S1 Fig Trem2 deficiency affects the number of decidual macrophages but not T cells and NK cells. (A) Representative flow cytogram of CD11b^+^ F4/80^+^ macrophages, comparing the absolute number of macrophages in wildtype and Trem2^-/-^ mouse placentas with or without *T*. *gondii* infection. The data were based on analysis conditions in Fig 2C. The placentas of each mouse were divided into three groups for technically repeated experiments. Data point represents the placenta of a single pregnant mouse (n = 6–8 mice). (B) A representative image of flow cytometry gating strategy for T cells and NK cells in mouse placentas. (C) Representative flow cytogram of T cells (CD45^+^ CD3^+^), comparing T cell proportions in wildtype and Trem2^-/-^ mouse placentas with or without *T*. *gondii* infection. The placentas of each mouse were divided into three groups for technically repeated experiments. Data point represents the placenta of a single pregnant mouse (n = 4 mice). (D) Representative flow cytogram of NK cells (CD45^+^ NK1.1^+^), comparing NK cell proportions in wildtype and Trem2^-/-^ mouse placentas with or without *T*. *gondii* infection. The placentas of each mouse were divided into three groups for technically repeated experiments. Data point represents the placenta of a single pregnant mouse (n = 4 mice). Data were presented as mean ± SD. Statistical analysis was conducted using one-way ANOVA with Tukey’s multiple comparisons test (A, C, and D). *: *P* < 0.05.
(TIF)

S2 Fig Trem2 deficiency further augments M1 polarization in the placentas of *T*. *gondii-*infected mice. (A) mRNA levels of CD86, CD206, iNOS and Arg1 in the mouse placentas were assayed by real-time PCR (n = 4 mice). (B) Representative flow cytogram of CD86^+^ (%) in CD11b^+^ BMDMs, comparing CD86 proportions in wildtype and Trem2^-/-^ BMDMs stimulated with or without *Tg*Ag. Data represent the results of three independent experiments. (C) Representative flow cytogram of CD206^+^ (%) in CD11b^+^ BMDMs, comparing CD206 proportions in wildtype and Trem2^-/-^ BMDMs stimulated with or without *Tg*Ag. Data represent the results of three independent experiments. Data were presented as mean ± SD. Statistical analysis was conducted using one-way ANOVA with Tukey’s multiple comparisons test (A, B and C). *: *P* < 0.05. *Tg*Ag: *T*. *gondii* antigens.
(TIF)

S3 Fig Trem2 deletion fails to affect the expression of NFκB, ERK, and mTOR. (A-C) NFκB, ERK, and mTOR protein levels in wildtype and Trem2^-/-^ mouse placentas with or without *T*. *gondii* infection were analyzed by immunoblot. Data point represents the placenta of a single pregnant mouse (n = 4 mice). Data were presented as mean ± SD. Statistical analysis was conducted using one-way ANOVA with Tukey’s multiple comparisons (A, B and C). *: *P* < 0.05.
(TIF)

S4 Fig Knockdown of Trem2 in THP-1 cells. THP-1 cells were transfected with siNC or three siTrem2 for 24 h. The expression of Trem2 protein was detected by immunoblot, and the statistical analysis was conducted by Image J. Data represent the results of three independent experiments. Data were presented as mean ± SD. Statistical analysis was conducted using one-way ANOVA with Tukey’s multiple comparisons test. *: *P*< 0.05.
(TIF)

S1 Table The sequences of siRNAs used in this study. (DOCX)

S2 Table The primer sequences used for real-time PCR in this study. (DOCX)
