# Supplementary figures and images for: Trem2/Syk/PI3K axis contributes to the host protection against Toxoplasma gondii-induced adverse pregnancy outcomes via modulating decidual macrophages
Source: PLoS Pathog. 2024 Sep 9;20(9):e1012543. doi: 10.1371/journal.ppat.1012543 (PMC11412541; doi:10.1371/journal.ppat.1012543)

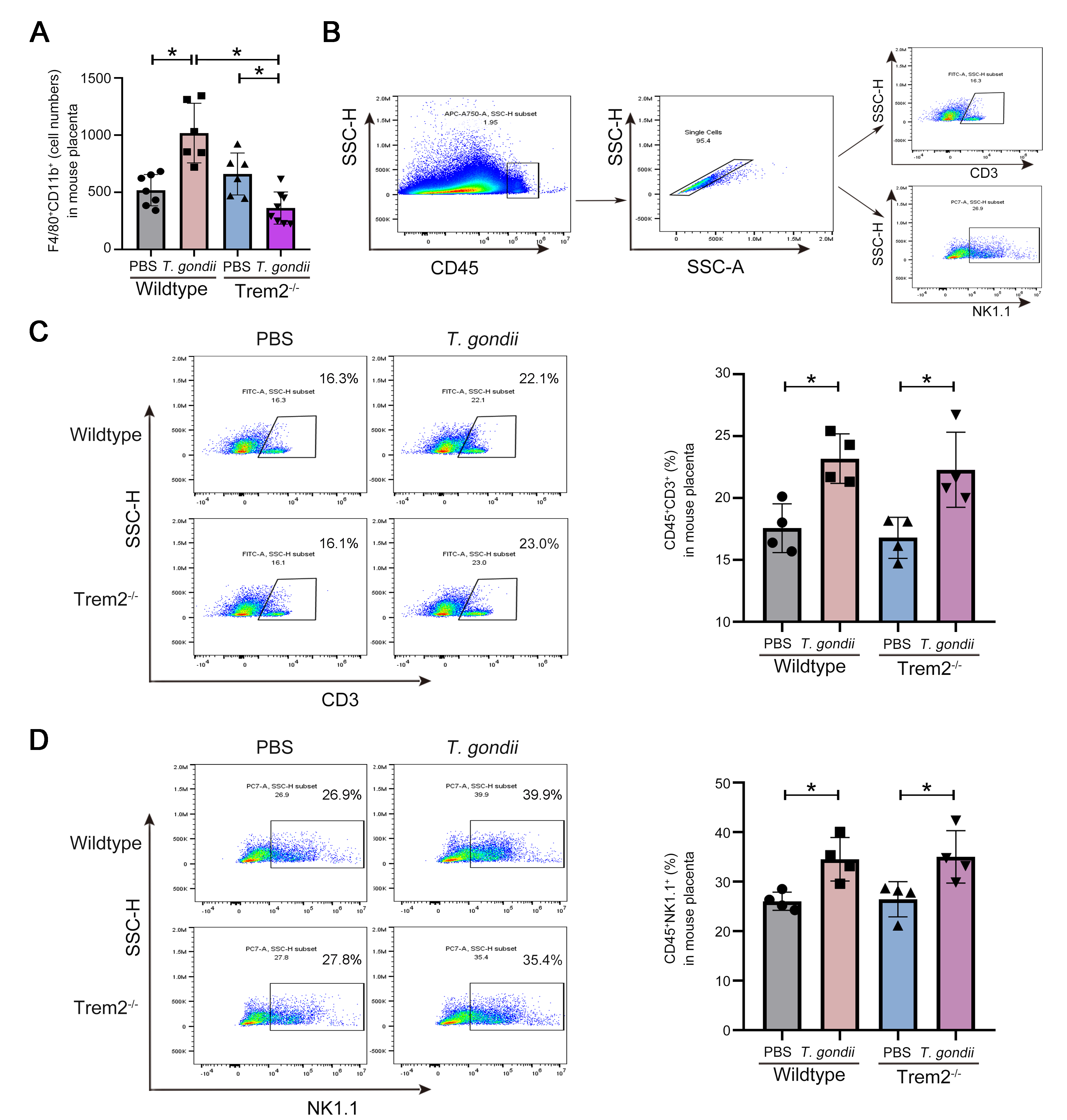

Supplement: S1 Fig — (A) Representative flow cytogram of CD11b+ F4/80+ macrophages, comparing the absolute number of macrophages in wildtype and Trem2-/- mouse placentas with or without T. gondii infection. The data were based on analysis conditions in Fig 2C. The placentas of each mouse were divided into three groups for technically repeated experiments. Data point represents the placenta of a single pregnant mouse (n = 6–8 mice). (B) A representative image of flow cytometry gating strategy for T cells and NK cells in mouse placentas. (C) Representative flow cytogram of T cells (CD45+ CD3+), comparing T cell proportions in wildtype and Trem2-/- mouse placentas with or without T. gondii infection. The placentas of each mouse were divided into three groups for technically repeated experiments. Data point represents the placenta of a single pregnant mouse (n = 4 mice). (D) Representative flow cytogram of NK cells (CD45+ NK1.1+), comparing NK cell proportions in wildtype and Trem2-/- mouse placentas with or without T. gondii infection. The placentas of each mouse were divided into three groups for technically repeated experiments. Data point represents the placenta of a single pregnant mouse (n = 4 mice). Data were presented as mean ± SD. Statistical analysis was conducted using one-way ANOVA with Tukey’s multiple comparisons test (A, C, and D). *: P < 0.05. (TIF) [file ppat.1012543.s001.tif]

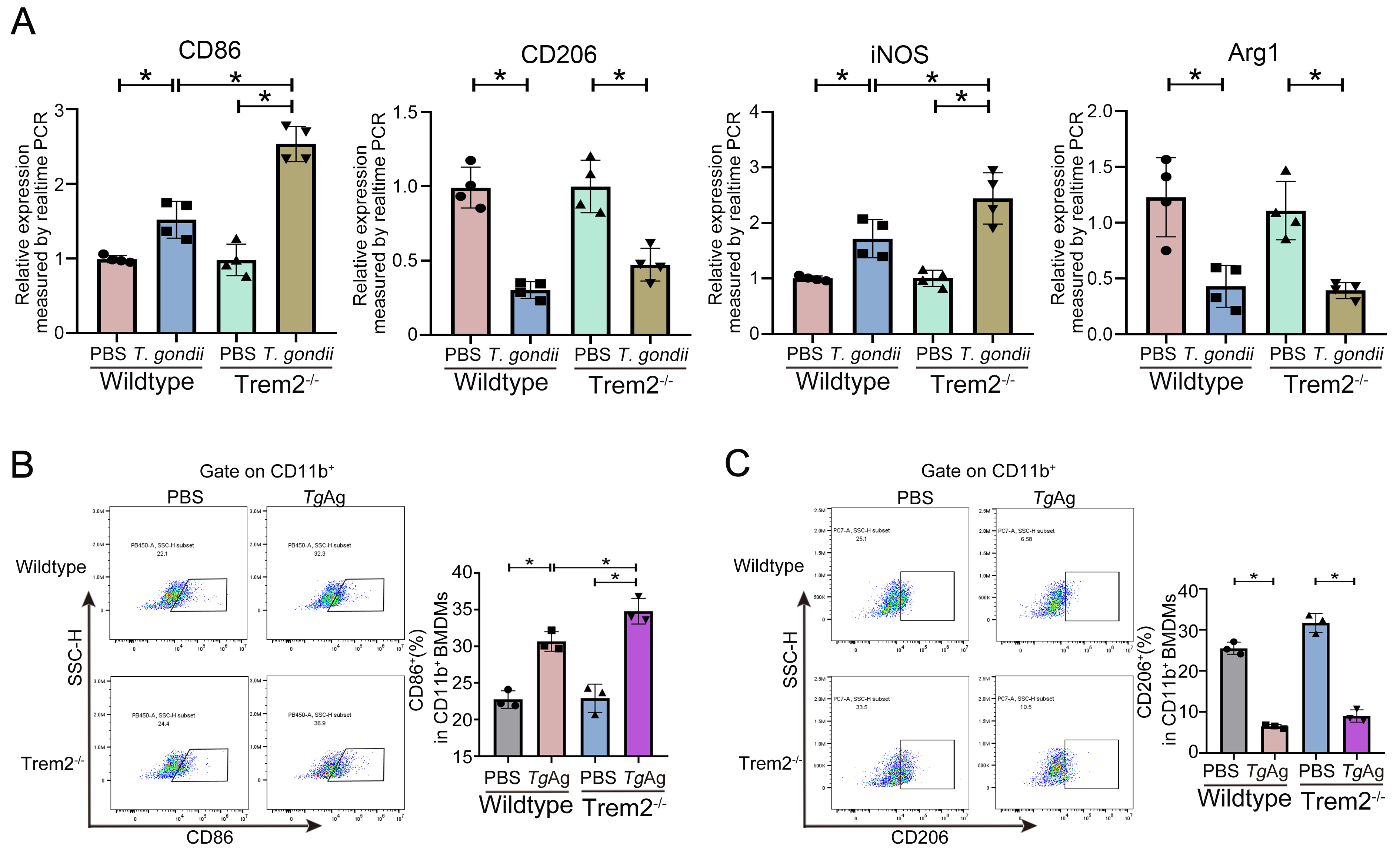

Supplement: S2 Fig — (A) mRNA levels of CD86, CD206, iNOS and Arg1 in the mouse placentas were assayed by real-time PCR (n = 4 mice). (B) Representative flow cytogram of CD86+ (%) in CD11b+ BMDMs, comparing CD86 proportions in wildtype and Trem2-/- BMDMs stimulated with or without TgAg. Data represent the results of three independent experiments. (C) Representative flow cytogram of CD206+ (%) in CD11b+ BMDMs, comparing CD206 proportions in wildtype and Trem2-/- BMDMs stimulated with or without TgAg. Data represent the results of three independent experiments. Data were presented as mean ± SD. Statistical analysis was conducted using one-way ANOVA with Tukey’s multiple comparisons test (A, B and C). *: P < 0.05. TgAg: T. gondii antigens. (TIF) [file ppat.1012543.s002.tif]

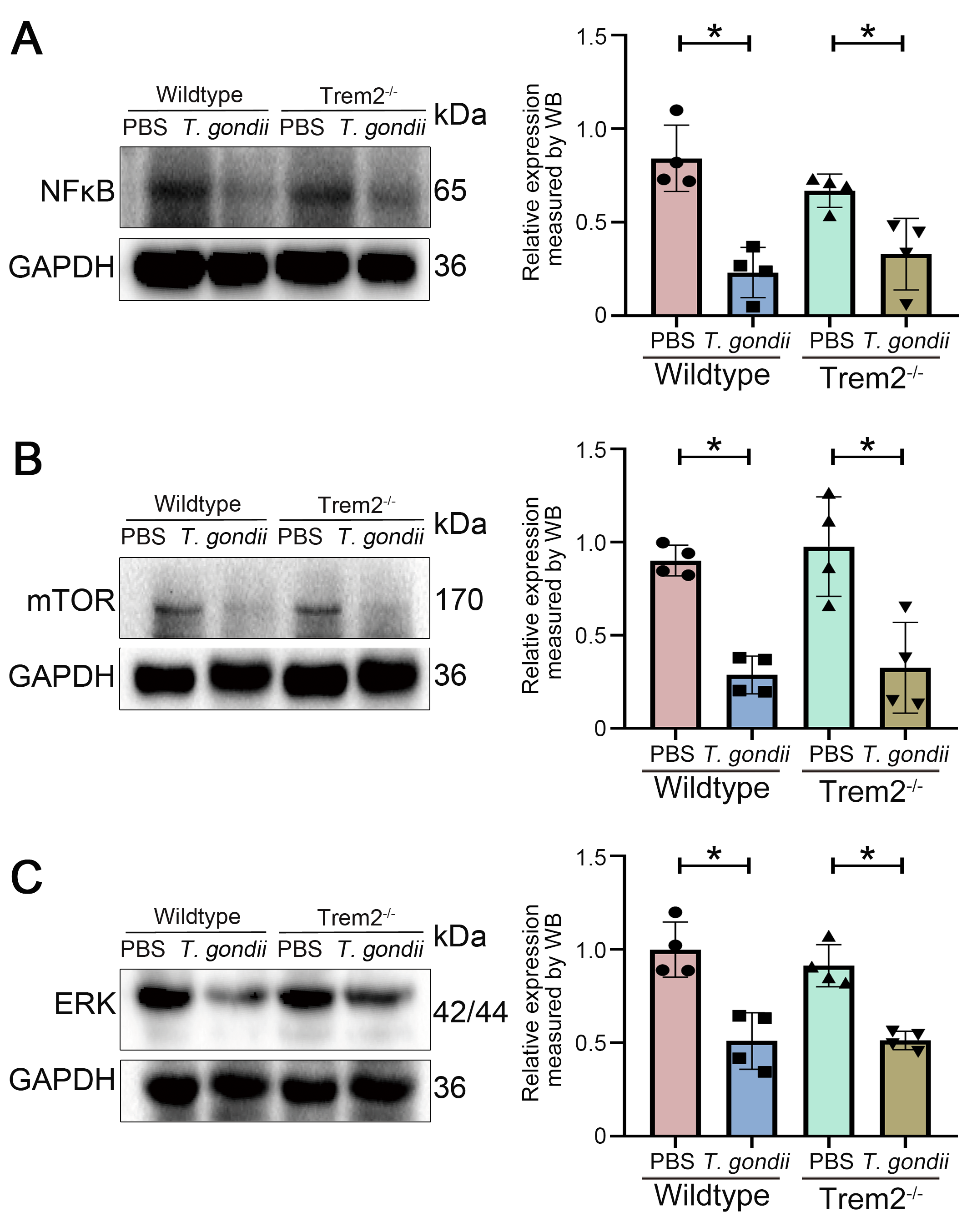

Supplement: S3 Fig — (A-C) NFκB, ERK, and mTOR protein levels in wildtype and Trem2-/- mouse placentas with or without T. gondii infection were analyzed by immunoblot. Data point represents the placenta of a single pregnant mouse (n = 4 mice). Data were presented as mean ± SD. Statistical analysis was conducted using one-way ANOVA with Tukey’s multiple comparisons (A, B and C). *: P < 0.05. (TIF) [file ppat.1012543.s003.tif]

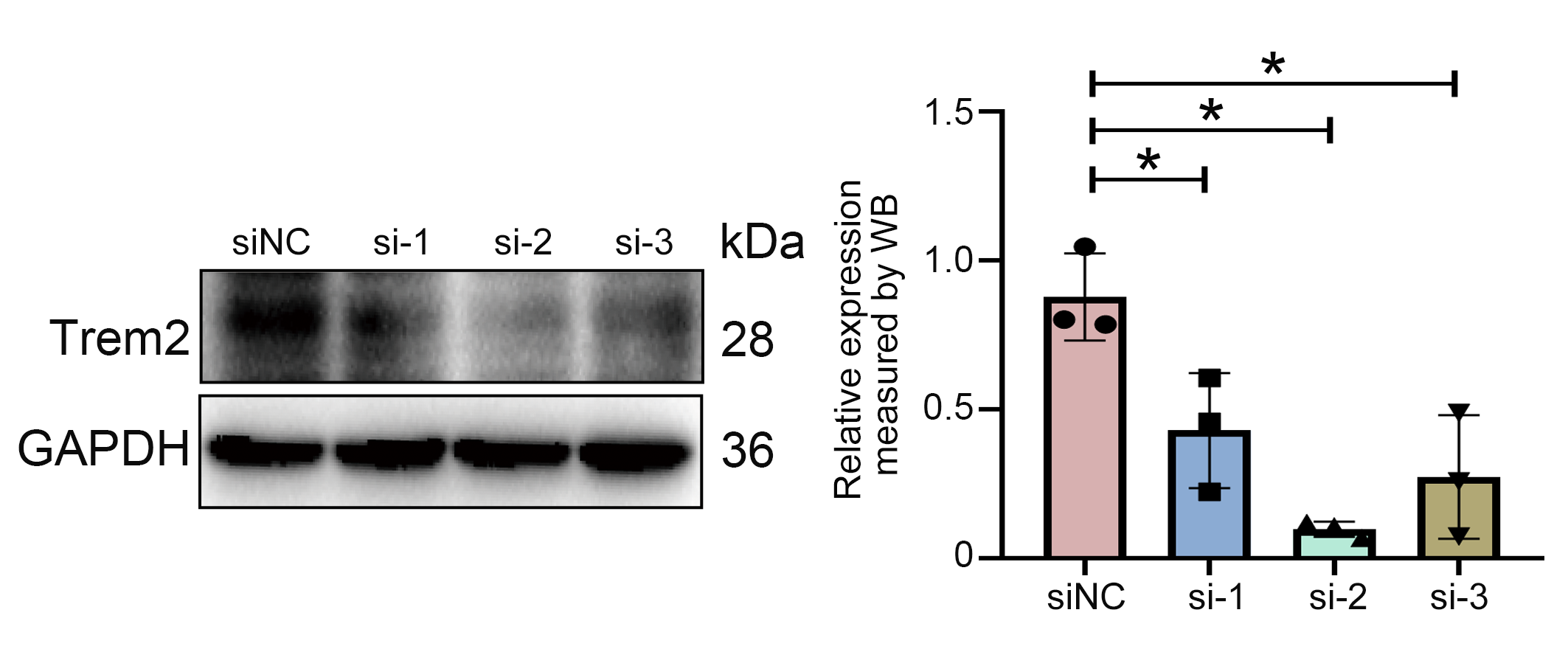

Supplement: S4 Fig — THP-1 cells were transfected with siNC or three siTrem2 for 24 h. The expression of Trem2 protein was detected by immunoblot, and the statistical analysis was conducted by Image J. Data represent the results of three independent experiments. Data were presented as mean ± SD. Statistical analysis was conducted using one-way ANOVA with Tukey’s multiple comparisons test. *: P< 0.05. (TIF) [file ppat.1012543.s004.tif]
