# Supplementary material for: Trem2/Syk/PI3K axis contributes to the host protection against Toxoplasma gondii-induced adverse pregnancy outcomes via modulating decidual macrophages
Source: PLoS Pathog. 2024 Sep 9;20(9):e1012543. doi: 10.1371/journal.ppat.1012543 (PMC11412541; doi:10.1371/journal.ppat.1012543)
Supplement: S1 Table — (DOCX) [file ppat.1012543.s005.docx]

**Table S1. The sequences of siRNAs used in this study.**

| siRNA | siRNA sequence (sense 5′-3′) | siRNA sequence (anti-sense 5′-3′) |
| --- | --- | --- |
| siTrem2-1 | GCUGCUCAUCUUACUCUUUTT | AAAGAGUAAGAUGAGCAGCTT |
| siTrem2-2 | CCAGGGUAUCAGCUCCAAATT | UUUGGAGCUGAUACCCUGGTT |
| siTrem2-3 | GGUGGCACUCUCACCAUUATT | UAAUGGUGAGAGUGCCACCTT |
| siNC | UUCUCCGAACGUGUCACGUTT | ACGUGACACGUUCGGAGAATT |
