# Supplementary material for: Trem2/Syk/PI3K axis contributes to the host protection against Toxoplasma gondii-induced adverse pregnancy outcomes via modulating decidual macrophages
Source: PLoS Pathog. 2024 Sep 9;20(9):e1012543. doi: 10.1371/journal.ppat.1012543 (PMC11412541; doi:10.1371/journal.ppat.1012543)
Supplement: S2 Table — (DOCX) [file ppat.1012543.s006.docx]

**Table S2. The primer sequences used for real-time PCR in this study**

| Primer names | Sequences |
| --- | --- |
| GAPDH F | TGGAAAGCTGTGGCGTGAT |
| GAPDH R | TGCTTCACCACCTTCTTGAT |
| GAPDH F (*T. gondii*) | CTCATTGTCGGCGGAAAGG |
| GAPDH R (*T. gondii*) | GGTGGACTCGCAGATGTAGTGG |
| CXCL1 F | CTGGGATTCACCTCAAGAACATC |
| CXCL1 R | CAGGGTCAAGGCAAGCCTC |
| CXCL5 F | GTTCCATCTCGCCATTCATGC |
| CXCL5 R | GCGGCTATGACTGAGGAAGG |
| G-CSF F | ATGGCTCAACTTTCTGCCCAG |
| G-CSF R | CTGACAGTGACCAGGGGAAC |
| IL-1β F | GCAACTGTTCCTGAACTCAACT |
| IL-1β R | ATCTTTTGGGGTCCGTCAACT |
| IL-6 F | ACAAAGCCAGAGTCCTTCAGAGA |
| IL-6 R | CTGTTAGGAGAGCATTGGAAATTG |
| TGF-β F | CGGTGCTCGCTTTGTA |
| TGF-β R | GCCACTCAGGCGTATC |
| IL-10 F | GCTCTTACTGACTGGCATGAG |
| IL-10 R | CGCAGCTCTAGGAGCATGTG |
| TNF-α F | AGGCACTCCCCCAAAAGATG |
| TNF-α R | CCACTTGGTGGTTTGTGAGTG |
| IL-12 F | TGGTTTGCCATCGTTTTGCTG |
| IL-12 R | ACAGGTGAGGTTCACTGTTTCT |
| IFN-γ F | ATGAACGCTACACACTGCATC |
| IFN-γ R | CCATCCTTTTGCCAGTTCCTC |
| CD86 F | TGTTTCCGTGGAGACGCAAG |
| CD86 R | TTGAGCCTTTGTAAATGGGCA |
| CD206 F | CTCTGTTCAGCTATTGGACGC |
| CD206 R | CGGAATTTCTGGGATTCAGCTTC |
| iNOS F | GTTCTCAGCCCAACAATACAAGA |
| iNOS R | GTGGACGGGTCGATGTCAC |
| Arg1 F | CTCCAAGCCAAAGTCCTTAGAG |
| Arg1 R | AGGAGCTGTCATTAGGGACATC |
